# A randomized controlled trial of a transdiagnostic cognitive-behavioral intervention for Afro-descendants’ survivors of systemic violence in Colombia

**DOI:** 10.1371/journal.pone.0208483

**Published:** 2018-12-10

**Authors:** Francisco J. Bonilla-Escobar, Andrés Fandiño-Losada, Diana M. Martínez-Buitrago, Julián Santaella-Tenorio, Daniel Tobón-García, Edgar J. Muñoz-Morales, Ivan D. Escobar-Roldán, Lori Babcock, Eva Duarte-Davidson, Judith K. Bass, Laura K. Murray, Shannon Dorsey, Maria I. Gutierrez-Martinez, Paul Bolton

**Affiliations:** 1 Instituto CISALVA, Universidad del Valle, Cali, Colombia; 2 Institute for Clinical Research Education, University of Pittsburgh, Pittsburgh, PA, United States of America; 3 SCISCO Foundation, Cali, Colombia; 4 Public Health School, Universidad del Valle, Cali, Colombia; 5 Epidemiology Department, Mailman School of Public Health, Columbia University, New York, NY, United States of America; 6 Psychiatry Residency Program, Department of Psychiatry and Behavioral Sciences, Duke University School of Medicine, Durham, NC, United States of America; 7 Heartland Alliance International, Chicago, IL, United States of America; 8 Department of International Health and Department of Mental Health, Bloomberg School of Public Health, Johns Hopkins University, Baltimore, MD, United States of America; 9 Department of Psychology, University of Washington. Seattle, WA, United States of America; Florida State University, UNITED STATES

## Abstract

**Background:**

Exposure to violence has negative consequences on mental health. Armed-conflict in Colombia has widely affected Afro-descendants in the Pacific region. Evidence regarding effectiveness of mental health interventions is lacking in low-income settings, especially in areas with active conflict. The objective of this study is to evaluate an individualized Common Elements Treatment Approach (CETA), a transdiagnostic psychotherapy model based on Cognitive-Behavioral Therapy, for adult trauma survivors.

**Methods and findings:**

A referred sample of 521 adult Afro-descendants from Buenaventura and Quibdó, Colombia, experiencing significant sadness, suffering or fear (score>0.77 in Total Mental Health Symptoms), with history of traumatic experiences, and with associated functional impairment were randomly allocated to CETA intervention, standby group without intervention, but under monthly monitoring, or a Narrative Community-Based Group Therapy. CETA was provided by trained Lay Psychosocial Community Workers without previous mental health experience, supervised by psychologists, during 12–14 weekly, 1.5-hour sessions. Symptoms were assessed with a locally validated survey built based on the Hopkins Symptom Checklist, the Harvard Trauma Questionnaire, the PTSD CheckList–Civilian Version, a qualitative study for additional general symptoms and a gender-specific functional impairment scale. CETA was compared with the control group and the intervention effects were calculated with mixed models using intention to treat analysis. Participant completion of follow-up was 75.1% and 13.2% voluntarily withdrew. Reduction in post-traumatic stress symptoms was significant in both municipalities when comparing intervention and control groups (mean difference), with a with a moderate effect size in Buenaventura (Cohen's d  =  0.70) and a small effect size in Quibdó (d = 0.31). In Buenaventura, the intervention also had significant effects on depression (large effect size d = 1.03), anxiety (large effect size d = 0.80) and functional impairment (moderate effect size d = 0.70). In Quibdó, it had no significant effect on these outcomes. Changes in Total Mental Health Symptoms were not significant in neither city.

**Conclusions:**

This trial suggests that CETA, can be effective in improving depression, anxiety, post-traumatic stress and function among victims of systematized violence in low-income and active conflict settings. Nonetheless, the difference of effectiveness between the two cities of intervention may indicate that we cannot assume that a mental health intervention known to be effective in one setting will be effective in another, even in similar circumstances and population. This may have special importance when implementing and reproducing these types of intervention in non-controlled circumstances. Further research should address these concerns. Results can be of use by governmental decision-makers when defining mental health programs for survivors.

**Trial registration:**

ClinicalTrials.gov NCT01856673 (https://clinicaltrials.gov/ct2/show/NCT01856673).

## Introduction

Colombia is ranked 2^nd^ in the world for the largest population experiencing internal displacement due to conflict and violence,[1] with a total of 8,873,105 internally displaced persons (IDPs) officially registered by the Colombian State’s Victims Unit (Sep, 2018),[2] and Afro-Colombians account for nearly 40%.[1, 3] Mental health conditions caused by displacement and associated violence often ends up unidentified and unattended.[4, 5] The Colombian Pacific is an area with large numbers of Afro-Colombian victims of the conflict.[6] There is limited information about displacement in Colombia including the average length of displacement, the different traumatic experiences suffered by victims of the armed conflict, or the effect of interventions on mental health of victims.[7–9] Previous research has shown that Afro-Colombian victims of violence experience symptoms of post-traumatic stress disorder (PTSD), depression, and anxiety.[9–13] Despite this, few psychosocial services are readily available for survivors of the conflict.[14] The importance and need for these services only gained some recognition and attention from the State after a Victims Law (No. 1448) was passed in 2011. Even so, programs targeting this population are still incipient and show strategic and methodological weaknesses (e.g.: Governmental Program for Attention to the Psychosocial and Holistic Health of Victims [PAPSIVI]).[15]

The two biggest cities of the Colombian Pacific region, a historically impoverished area, are Buenaventura, a harbor city in Valle del Cauca province (department) and Quibdó, the capital of Chocó province. In both cities there are large concentrations of Afro-Colombian IDPs.[16] Although several processes of disarmament of militia have taken place over the last three decades, and a peace agreement with the country’s largest armed group, the Revolutionary Armed Forces of Colombia (FARC), violence reduction has been marginal due to the presence of other criminal groups.[1, 17] A qualitative study carried out in 2011 with Afro-Colombians in these two communities found that the predominant problems for victims were those related to trauma, violence, displacement and poverty and that the most prevalent mental health symptoms included anxiety, depression and fear.[12]

The current study is part of a larger five-year program called “Community-Based Treatment Services for Afro-Colombian Victims of Conflict and Torture” (ACOPLE), financed by the United States Agency for International Development (USAID). Heartland Alliance International (HAI) and the National Association of Displaced Afro-Colombians (AFRODES) implemented ACOPLE. Two different interventions were employed and evaluated: the individually administered Common Elements Treatment Approach (CETA) [18] described in this paper and a Narrative Community-Based Group Therapy (NCGT), described in a separate manuscript. This decision was based on the differences between the interventions including their standpoints, aims, and methodologies.[8, 9] The CISALVA Institute of Universidad del Valle, Colombia, led these processes with the technical support by faculty at Johns Hopkins Bloomberg School of Public Health. The aim of the study described in this paper was to evaluate the effectiveness of CETA in Afro-Colombians survivors of violence. Two prior randomized controlled trials testing CETA compared to waitlist control in Southern Iraq,[19] and on the Thai-Burma border,[20] demonstrated strong effectiveness of CETA for post-traumatic stress and depression (large effect sizes).

Transdiagnostic approaches in the treatment of common mental health problems have been tested and found useful in different populations in the United States [21, 22] and in other global settings.[20, 23] In this case, a community-based approach, considered as such due to delivery of the intervention by lay psychosocial community workers (LPCW) from the same community, was chosen as opposed to being delivered by mental health professionals. In other countries, it has proved to be an effective alternative in areas where there are few professionals available. This was considered as the easiest way to access victims, promoting confident relationships between participants and LPCW.[7, 23–25]

To our knowledge, there are not any published trials evaluating mental health interventions provided by non-mental health professionals for victims of violence in Latin America.

## Materials and methods

### Settings and population

Initial recruitment of participants was done using referral from key informants (AFRODES community leaders) who were able to contact people affected by violence and displayed suffering, sadness or symptoms of depression in selected communities of both cities.[26] Key informants were requested to refer both men and women, followed by a snowball technic where the interviewed were asked to suggest others that might have presented the same conditions. Other individuals were identified by visiting neighborhoods with a high prevalence of victims.

The trial was described to potential study participants; informed consent was explained and signed prior to baseline assessment, selection, and randomization. Inclusion criteria were: a) being over 18 years old, b) identifying as Afro-Colombian, c) reporting having experienced at least one violent traumatic experience, d) having a TMHS (Total Mental Health Symptoms Scale) score ≥0.77 (49 out of 64 items), and e) having any reduced functionality in routine activities. All those endorsing or having suicidal thoughts and requiring psychological or medical care, as determined by psychologists, were excluded and psychological treatment was provided or they were referred to local health services. As the provision of mental health care was limited in the cities,[27] ACOPLE implemented one center in each city where there was one psychologist full-time. These psychologists were in charge of provision of care to the excluded clients, to those in crisis, to help the helper, and to the controls when they finished their follow-up period. Relatives to the LPCW were also excluded to avoid contamination between the arms and the introduction of bias that can affect the recommendations from the LPCW to the clients. None of the volunteers received psychiatric pharmacotherapy before or during the study.

Consecutive ID numbers were assigned in a masked manner to baseline assessment surveys, which were conducted by trained social work students who did not participate either in the randomization or in the interventions. Only those subjects over the inclusion cut-off score were included in the randomization process. Local researchers of CISALVA, who had not access to participants’ assessment results, did the masked random allocation of participants using a blocked randomization procedure. Blocks of 6 cells were randomized to allocate participants in the three arms, the two interventions of ACOPLE (CETA and NCGT) and the control group (two cells of each arm were also randomly distributed per block). Subjects were assigned to one cell of the block in the order they finished they baseline survey. This process of allocation of participants continued until the calculated sample size was reached. The randomization remained masked until the subjects’ collection was concluded. The interviewers who conducted the baseline and follow-up assessments were masked, Participants were able to recognize the arm in which they had been allocated once the interventions had started.

One psychologist accompanied interviewers during surveys to attend any psychological crisis that arose. Trained interviewers in people’s homes or local community centers (e.g., schools and churches) conducted baseline assessments. In Buenaventura, most surveys were conducted in a local church for security reasons. Follow-up assessments were conducted in the ACOPLE centers, people’s homes, or in a local church. Some consented participants allocated to the intervention arm were not able to immediately receive the services for 8 or more weeks due to restricted capacities of LPCW. For them a second baseline assessment was conducted before initiation of the intervention (n = 10 in Quibdó; n = 8 in Buenaventura) and considered as the baseline. The follow-up assessment was conducted approximately two weeks after completion of the therapy for intervention participants. Follow-up assessments for controls were conducted, approximately, at 3–4 months after baseline assessment according to their time of enrollment.

The control participants were unlikely to receive any treatment outside the study as access to these kinds of services are extremely limited in both cities.[27] Controls were contacted by study staff on a monthly basis to screen for acute serious mental problems such as suicide ideation, anxiety, or depression. The participants in the control group were assessed by a psychologist and, when necessary, they were excluded from the study and provided with the appropriate care including psychological care or a referral to a local psychiatry who visits the cities once or twice every month. A psychological evaluation was offered and provided to each of the controls after their completion of the follow-up and, when required, psychological care was provided in ACOPLE centers.

Using data from a CETA pilot study conducted before the trial, where a 0.3 points reduction of the mean changes scores from baseline to follow-up (20% of the whole scale) was found (according to the Total Mental Health Symptoms Scale, TMHS), a standard deviation (SD) of 0.47 points in both groups and statistical power of 80% (two sided alpha at 0.05), we calculated a sample size of 39 people per arm in each municipality for comparing the CETA intervention and control group. We considered a 20% attrition rates therefore the expected sample size was 47 subjects per group.

### Instruments and outcome assessment

An instrument validation study, conducted with a sample of Afro-Colombians from the two study communities, was carried out in 2011, to evaluate the psychometric properties of the culturally adapted mental health and dysfunction assessments. In addition, the questionnaire included socio-demographic, socioeconomic, traumatic experiences, access to healthcare services, and support networks variables (*Study Protocol* DOI: dx.doi.org/10.17504/protocols.io.pgvdjw6).

Mental health symptoms during the previous month were measured with the TMHS scale of 64 items, ranging from 0 for “never” to 3 for “all the time”, including locally relevant symptoms and sub-scales of depression (n = 15 symptoms), anxiety (n = 10 symptoms) and post-traumatic stress symptoms (PTSS) (n = 16 symptoms). The primary outcome was the change on TMHS and secondary outcomes were changes in depression, anxiety, PTSS, and dysfunction scales. Symptoms of depression, anxiety, and trauma (PTSS) were assessed with a validated instrument built based on the Hopkins Symptom Checklist (HSCL-25),[28] the Harvard Trauma Questionnaire (HTQ),[29] the PTSD CheckList–Civilian Version (PCL-C),[30] and a qualitative study where general symptoms and other qualitative variables were identified. The instruments were translated into Spanish using the language and terminology from the previous qualitative study and included some local and/or culturally appropriate terms to ensure local understanding. In both cities the TMHS showed high internal consistency (Cronbach’s alpha was 0.95 in Quibdó and 0.96 in Buenaventura). The internal consistency was also high for the scales, with depression, anxiety and PTSD Cronbach’s alpha of 0.84, 0.88, and 0.85 in Quibdó, and 0.85, 0.88, 0.89 in Buenaventura, respectively (S1 File. Validating an instrument for victims of violence in Colombia). The symptom scales were used to characterize severity of the study participant’s psychological symptoms.[31–33] The dysfunction measure was a gender-specific questionnaire with 12-items for females and 10-items for males. Each item assessed a task ranging from 0 for “no difficulty” to 4 for “cannot do it”.

Outcome assessment was done per participant by the calculation of items’ mean for each scale and sub-scales. Changes between pre- and post-intervention scores were calculated.

### Common Elements Treatment Approach (CETA)

LPCW were trained in the CETA intervention, which is a transdiagnostic psychotherapy model based on Cognitive-Behavioral Therapy developed by two of the authors (LM, SD) for use in low-resource contexts.[18] CETA was developed to treat post-traumatic stress, depression, and anxiety and other comorbid problems. Treatment duration is 8–12 sessions (45 min to 1 hour). The intervention is modular (meaning that elements can mostly stand alone and be delivered in any order). CETA LPCWs were trained both in nine components including: (1) Encouraging Participation, (2) Psycho-education, (3) Cognitive coping, (4) Gradual exposure: trauma memories, (5) Cognitive reprocessing, (6) Safety skills, (7) Relaxation, (8) Behavioral activation and (9) Live gradual exposure.[18] LPCWs were also trained in decision making for selecting elements, sequencing (e.g., which are needed first) elements, and dosing of elements (e.g., how many sessions of each element), based on a client’s symptom presentation and response to treatment. In the prior RCTs, CETA was delivered in an average of 9.7 sessions in the Thai-Burma study[20] and in 9.94 sessions in the Southern Iraq study.[19]

The LPCW were all Afro-Colombian survivors of violence and displacement, recognized leaders and/or caregivers in their receiving communities, and had at least five years of post-primary education. Buenaventura’s LPCW had more community-work experience, defined as number of years working in social projects, than those from Quibdó (8 and 7 years, respectively).

Twenty LPCW (10 from each city) were trained and supervised via the apprenticeship model of training.[18, 34] They participated in a 10-day training by CETA experts (LM, SD, and 2 others), followed by weekly practice groups and then personalized supervision meetings with a psychologist and a local supervisor (one per city) after each session.[26] This was important, not only to ensure fidelity to the CETA treatment, but also as a means to ensure alignment with the Norms and Codes of Practice of Psychology in Colombia.[35] Following the Apprenticeship model,[18] the local supervisor was supported by one of the CETA trainers through weekly 1–2 hour Skype calls. Skype calls involved simultaneous Spanish-English translation between the CETA expert and local supervisor.

Once the local supervisor and psychologist deemed that a LPCW was sufficiently trained to begin providing CETA, each LPCW started with only a “pilot” (non-study) client and their CETA provision was very closely supervised. LPCWs who managed their first client were able to take a second client. Once LPCWs demonstrated skill with two clients, they began to see study cases that were randomized to CETA. LPCWs met weekly with participants, providing treatment sessions in rudimentary buildings in each municipality, provided by HAI, where other therapies also were provided.

### Statistical methods

Baseline characteristics of participants from intervention and control groups were compared using Chi-square or two-tailed Fisher’s exact test for categorical variables and t-student or Wilcoxon test for continuous variables. Extreme values in scales (more or less 4 Standard Deviations) were identified as potential outliers. The indicators of effect derive from a comparison of the mean changes scale scores from baseline to follow-up assessment between intervention and control groups.

The design of the study was intended to have separated effect analyses in each municipality. Linear mixed-effects models were used to estimate the effect of treatment on mental health and dysfunction outcomes, defined as the interaction between treatment and time. This assessed the mean difference between treatment conditions (CETA vs. control) in changes of outcomes over time within each group. Two random-effects were evaluated: LPCWs and participant, being statistically relevant (p<0.001), thus, regression models were adjusted for both effects as a random term in the regression. Time, treatment condition, and their interaction were included as fixed effects. Statistical significance was set at the 0.05 alpha level. Co-variables included in final mixed-models were those that were significant at the p<0.10 level identified using: 1) simple logistic regression clustering by LPCWs to identify baseline differences between intervention and control groups; 2) mixed models to determine interactions between potential co-variables and time on symptoms and dysfunction scores. Furthermore, models were adjusted for age, gender, education and marital status, which are important confounders in psychological research.[36]

An Intention-to-treat (ITT) analysis, which included all study participants based on participants’ arm allocation when they were randomized, was used to mitigate potential selection bias by participants or interviewers. Preplanned sub-group analyses by marital status were conducted, considering that being married is an important protective factor of mental health illness.[37, 38] Exploratory post-hoc analyses were carried out about the number of sessions per city and their effect (dose-response) among CETA participants with follow-up using mixed-models adjusted by ITT covariates. Finally, sensitivity analyses were conducted excluding one LPCW each time to ascertain provider-based biases. Outlier participants (two in Quibdó) were excluded in a sensitivity analysis to evaluate its impact on intervention outcomes. Between group effect sizes were calculated using Cohen’s d statistic.[39, 40]

Stata version 15 (Stata Corp, College Station, TX, USA) was used for the statistical analyses. The study was approved by the Institutional Review Board, Human Ethics Committee of Universidad del Valle, Colombia (Code 014–011).

## Results

**Fig 1:**
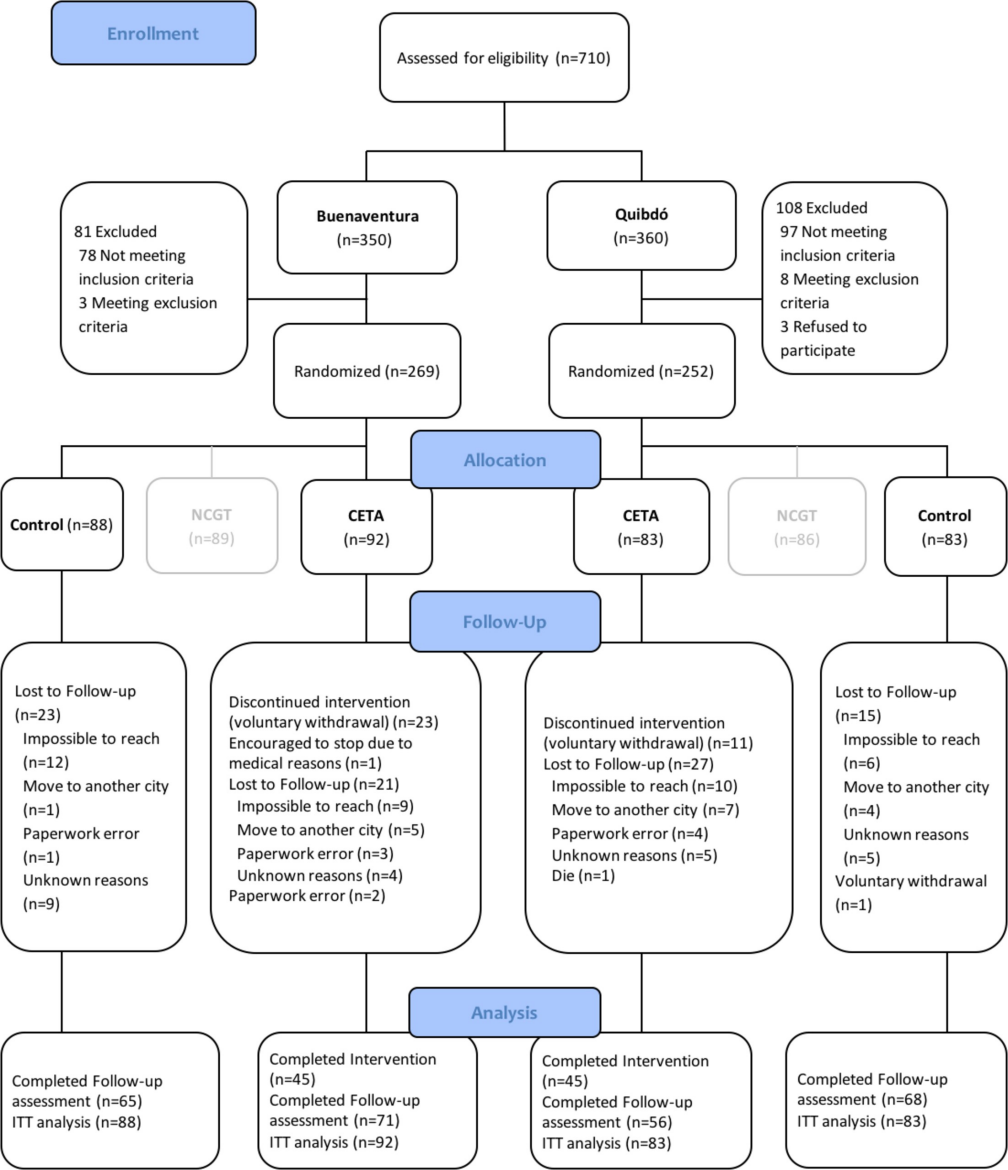
Flow chart of study participants.

A total of 710 eligibility assessments were conducted across study cities (n = 350 in Buenaventura and n = 360 in Quibdó) from May 2012 to September 2013. In Buenaventura, 180 participants were found eligible and allocated to the intervention subject to this paper, CETA (n = 92; 51.1%) or the control group (n = 88; 48.9%). In Quibdó, 166 participants were found eligible and allocated in equal numbers to each arm (n = 83 per arm). The remaining 89 and 86 randomized participants from Buenaventura and Quibdó, respectively, were allocated to the other intervention (NCGT) which results are reported elsewhere (https://clinicaltrials.gov/ct2/show/NCT01856673). A total of 260 participants completed the follow-up assessment (71 CETA and 65 controls in Buenaventura and 56 CETA and 68 controls in Quibdó) (Fig 1).

Overall, 34 participants withdrew voluntarily from the CETA intervention, most of them due to work and school schedule issues, one was encouraged to stop the treatment because of dementia according to psychological evaluation, 48 were lost to follow-up, one died for reasons unrelated to the study (in Quibdó), and two were excluded after randomization due to paperwork error.

### Socio-demographic and baseline characteristics

In Buenaventura and Quibdó, socio-demographic and socio-economic variables were similar between intervention and control groups (p>0.05). In Buenaventura, traumatic experiences score was significantly higher in CETA than in the control group (p = 0.04). In Quibdó, the mean TMHS and anxiety scores were significantly greater in the control group than in the CETA group (p = 0.04 and p = 0.05, respectively), and men dysfunction score was significantly higher in CETA than in control group (p = 0.05) (Table 1).

**Table 1:** Characteristics of trial participants and baseline scores.

Characteristics	Buenaventura			Quibdó		
	Control (n = 88)	CETA (n = 92)	p	Control (n = 83)	CETA (n = 83)	p
**Gender, n (%)**			0.71^a^			0.53^a^
Men	9 (45)	11 (55)		12 (44.4)	15 (55.6)	
Women	79 (49.4)	81 (50.6)		71 (51)	68 (48.9)	
**Age, mean (SD)**	41.8 (14.8)	40.4 (14.1)	0.52^b^	44 (18.9)	46 (19)	0.50^b^
**Marital status, n (%)**			0.66^a^			0.44^c^
Partnered	45 (47.4)	50 (52.6)		37 (46.3)	43 (53.7)	
Single	43 (50.6)	42 (49.4)		45 (52.9)	40 (47.1)	
Missing data	-	-		1 (100)	-	
**Education, n (%)**			0.43^a^			0.76^a^
No education	7 (38.9)	11 (61.1)		18 (46.1)	21 (53.9)	
Primary	39 (54.2)	33 (45.8)		37 (49.3)	38 (51.7)	
Secondary or above	42 (48.9)	48 (53.3)		28 (53.9)	24 (46.1)	
**Displaced, n (%)**			0.58^a^			1.00^c^
No	14 (54)	12 (46)		2 (50)	2 (50)	
Yes	74 (48)	80 (52)		81 (50)	81 (50)	
**Employment status, n (%)**			0.27^a^			0.69^a^
Employed	18 (56.3)	14 (43.7)		11 (42.3)	15 (57.7)	
Informal worker	18 (39.1)	28 (60.9)		15 (51.7)	14 (48.3)	
Unemployed	52 (51)	50 (49)		57 (51.4)	54 (28.6)	
**Main material in house flooring, n (%)**			0.54^a^			0.14^c^
Tile/Aniline/Brick/Cement	37 (47.4)	41 (52.5)		23 (43.4)	30 (56.6)	
Wood	22 (44)	28 (56)		22 (44)	28 (56)	
Tamped ground or other	29 (55.8)	23 (44.2)		37 (59.7)	25 (40.3)	
Missing data	-	-		1 (100)	-	
**Health care regime, n (%)**			0.08^c^			0.42^c^
Contributive	2 (28.6)	5 (71.4)		4 (33.3)	8 (66.7)	
Subsidized	78 (47.8)	85 (52.2)		75 (52.8)	67 (47.2)	
Not covered	8 (80)	2 (20)		3 (33.3)	6 (66.7)	
Missing data	-	-		1 (25)	2 (75)	
**Traumatic experiences, mean (SD)**	3.5 (1.6)	4 (1.6)	0.04^b^	4 (1.7)	3.6 (1.7)	0.13^b^
**Mean Scale scores, mean (SD)**						
Total Mental Health Symptoms	1.5 (0.5)	1.4 (0.5)	0.18^b^	1.5 (0.4)	1.3 (0.4)	0.04 ^b^
Depression Symptoms	1.6 (0.5)	1.5 (0.5)	0.18^b^	1.5 (0.5)	1.4 (0.5)	0.20^b^
Anxiety Symptoms	1.7 (0.6)	1.6 (0.7)	0.31^b^	1.7 (0.6)	1.5 (0.7)	0.05^b^
Post-trauma stress Symptoms	1.7 (0.5)	1.7 (0.5)	1.00^b^	1.7 (0.5)	1.6 (0.5)	0.20^b^
Women’s Dysfunction	0.9 (0.6)	0.9 (0.5)	1.00^b^	0.8 (0.6)	0.8 (0.7)	1.00^b^
Men’s Dysfunction	0.7 (0.5)	0.7 (0.5)	1.00^b^	0.8 (0.95)	1.1 (1.0)	0.05^b^

SD: Standard deviation

^a^ Chi-square test,

^**b**^ t test,

^**c**^ Fisher's exact test

### Effectiveness of the intervention

**Fig 2:**
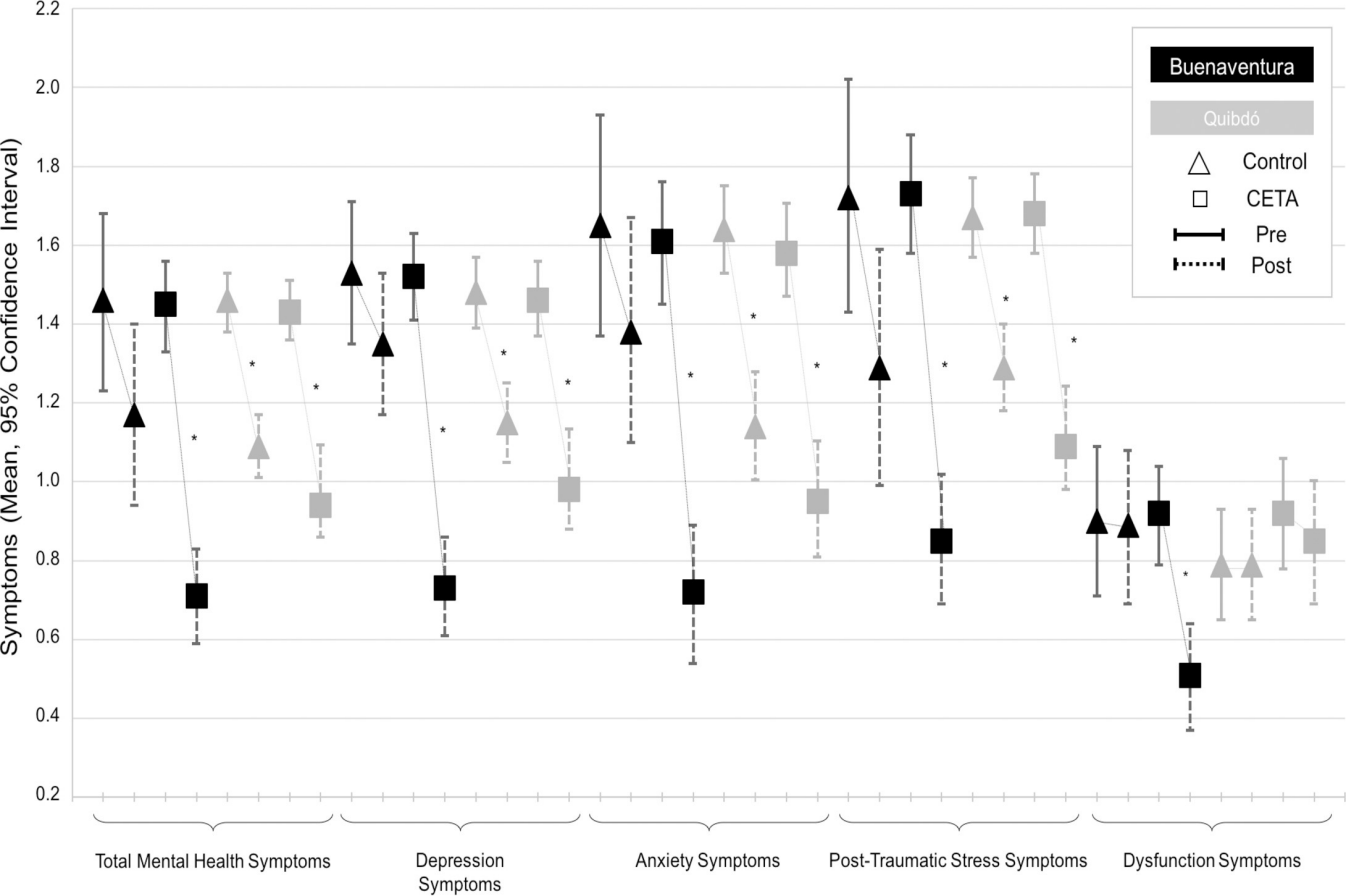
Adjusted pre and post symptom score means with their 95% confidence intervals by arm and city. *: Significant difference at p<0.05.

In Buenaventura, the adjusted comparison of baseline and follow-up assessments of symptoms in CETA showed significant differences compared with the changes in the control group. Meanwhile in Quibdó, the level of symptoms of both CETA and the control group were similar in the baseline and the follow-up assessments. Fig 2 presents the adjusted pre and post symptoms score means with their 95% confidence intervals by arm and city. In Buenaventura, all the control comparisons (baseline vs. follow-up) overlapped, in contrast, the CETA comparisons did not overlapped. In Quibdó, all the adjusted assessments of CETA and control arms behave similarly with significant differences between baseline and follow-up assessments in both groups except for the dysfunction scale (Table 2 and Fig 2).

**Table 2:** Assessment of the CETA effectiveness.

Scale/ Sub-scale	Mea-sure	Buenaventura				Quibdó			
		Control (95% CI)	CETA (95% CI)	p	Effect size	Control (95% CI)	CETA (95% CI)	p	Effect size
**Total Mental Health Symptoms**	**BL**	1.46 (1.23; 1.68)	1.45 (1.33; 1.56)			1.46 (1.38; 1.53)	1.43 (1.36; 1.51)		
	**FU**	1.17 (0.94; 1.40)	0.71 (0.59; 0.83)			1.09 (1.01; 1.17)	0.94 (0.86; 1.01)		
	**FU—BL**	-0.28 (-0.40; -0.17)	-0.74 (-0.85; -0.63)	<0.0001^a^	0.82	-0.37 (-0.48; -0.26)	-0.49 (-0.60; -0.39)	0.10^b^	0.22
	**Diff**	-0.46 (-0.61; -0.30)				-0.12 (-0.27; 0.02)			
**Depression**	**BL**	1.53 (1.35; 1.71)	1.52 (1.41; 1.63)			1.48 (1.39; 1.57)	1.46 (1.37; 1.56)		
	**FU**	1.35 (1.17; 1.53)	0.73 (0.61; 0.86)			1.15 (1.05; 1.25)	0.98 (0.88; 1.08)		
	**FU—BL**	-0.18 (-0.32; -0.04)	-0.79 (-0.92; -0.65)	<0.0001^c^	1.03	-0.33 (-0.46; -0.2)	-0.48 (-0.62; -0.35)	0.10^d^	0.27
	**Diff**	-0.61 (-0.80; -0.42)				-0.15 (-0.34; 0.03)			
**Anxiety**	**BL**	1.65 (1.37; 1.93)	1.61 (1.45; 1.76)			1.64 (1.53; 1.75)	1.58 (1.47; 1.70)		
	**FU**	1.38 (1.10; 1.67)	0.72 (0.54; 0.89)			1.14 (1.004; 1.28)	0.95 (0.81; 1.09)		
	**FU—BL**	-0.27 (-0.44; -0.09)	-0.89 (-1.06; -0.71)	<0.0001^e^	0.80	-0.5 (-0.67; -0.32)	-0.63 (-0.81; -0.45)	0.30^f^	0.20
	**Diff**	-0.62 (-0.87; -0.38)				-0.13 (-0.38; 0.12)			
**Post-trauma stress**	**BL**	1.72 (1.43; 2.02)	1.73 (1.58; 1.88)			1.67 (1.57; 1.77)	1.68 (1.58; 1.78)		
	**FU**	1.29 (0.99; 1.59)	0.85 (0.69; 1.02)			1.29 (1.18; 1.4)	1.09 (0.98; 1.2)		
	**FU—BL**	-0.43 (-0.58; -0.28)	-0.88 (-1.02; -0.73)	<0.0001^g^	0.70	-0.38 (-0.53; -0.23)	-0.59 (-0.74; -0.44)	0.053^h^	0.31
	**Diff**	-0.45 (-0.66; -0.24)				-0.21 (-0.42; 0.002)			
**Dysfunction**	**BL**	0.90 (0.71; 1.09)	0.92 (0.79; 1.04)			0.79 (0.65; 0.93)	0.92 (0.78; 1.06)		
	**FU**	0.89 (0.69; 1.08)	0.51 (0.37; 0.64)			0.79 (0.65; 0.93)	0.85 (0.69; 1.003)		
	**FU—BL**	-0.011 (-0.17; 0.15)	-0.41 (-0.56; -0.26)	<0.0001^i^	0.70	-0.001 (-0.17; 0.17)	-0.08 (-0.26; 0.1)	0.55^j^	0.12
	**Diff**	-0.40 (-0.62; -0.18)				-0.08 (-0.33; 0.18)			

Mixed effect clustered models with multiple chained imputations. All the models were adjusted by age, gender, marital status, education, employment status, type of floor at home, type of health coverage, baseline TMHS, traumatic experiences, baseline assessment of sadness, baseline assessment of suffering, and time on study. LPCW and participant were used cluster variables.

Additional adjusting variables include:

a) displaced condition, past psychological support, functionality at baseline;

b) number of people from which you can borrow a small amount of money;

c) displaced condition, past psychological support, number of people from which you can borrow a small amount of money, baseline functionality;

d) number of people from which you can borrow a small amount of money;

e) displaced condition, past psychological support, number of people from which you can borrow a small amount of money, past psychological support, baseline functionality;

f) displaced condition, number of people from which you can borrow a small amount of money;

g) displaced condition, past psychological support, number of people from which you can borrow a small amount of money, past psychological support, baseline functionality;

h) number of people from which you can borrow a small amount of money;

i) number of people cohabitating, and number of people from which you can borrow a small amount of money;

j) number of people from which you can borrow a small amount of money.

95% CI: 95% confidence interval. BL: Baseline symptom score, mean. FU: Follow-up symptom score, mean. FU—BL: Difference from baseline to follow-up, mean. Diff: Difference in adjusted mean score change, mean. The between group effect size was measured with Cohen´s d ([mean of group 1—mean of group 2] /pooled standard deviations for the two groups) Effect size interpretation: 0.20–0.49: Small effect; 0.50–0.79: Moderate effect; ≥0.80: large effect.

**Fig 3:**
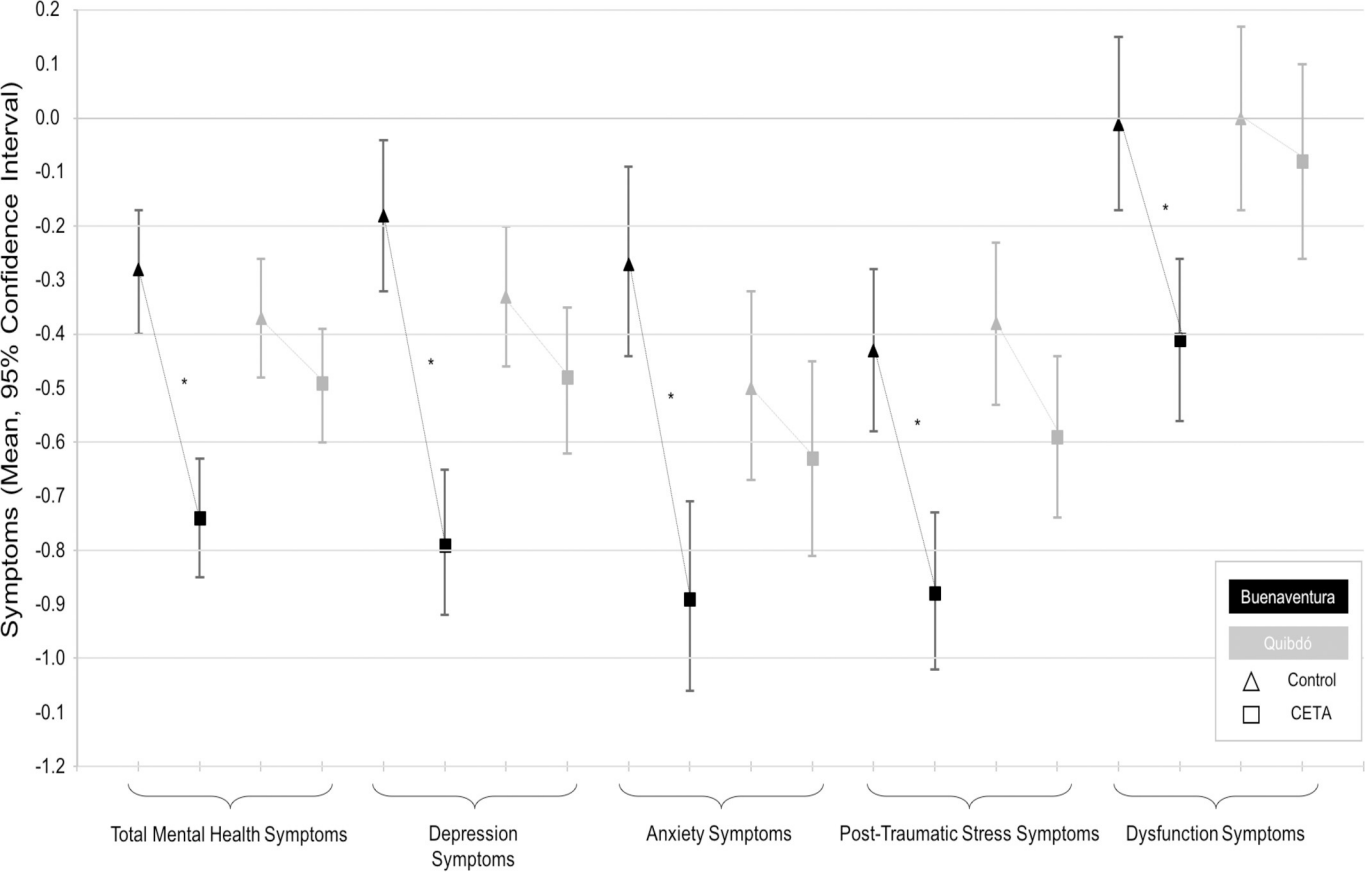
Adjusted pre-post differences of symptoms scores with their 95% confidence intervals by arm and city. *: Significant difference at p<0.05.

Table 2 presents the intervention effects for all outcomes across both cities measured at baseline, follow-up, the difference between the follow-up and the baseline, and the difference of the interventions comparing the difference of follow-up and baseline (difference of the differences). In Buenaventura, participants in the CETA intervention experienced, on average, larger effects across all outcomes compared with control participants. In Quibdó, small effects were seen only for improvement in the PTSS scale score (Fig 3). Sensitivity analyses, removing Quibdó outliers, increased the significance of CETA effect in the PTSS scale (-0.22, 95% Confidence interval (95%CI): -0.42;-0.009, p = 0.04).

Sub-group analyses showed a large effect in people who had a partner for all scales and subscales in Buenaventura and moderate effect for depression and PTSS in Quibdó (Table 3).

**Table 3:** Sub-group analysis of the effect of CETA among Afro-Colombian victims of violence in the Pacific region of Colombia.

Scale/Sub-scale	Buenaventura		Quibdó	
	Mean difference (95% CI)	Effect size	Mean difference (95% CI)	Effect size
**Total Mental Health Symptoms**				
Single	-0.38 (-0.61; -0.15) ^§^	0.70	-0.06 (-0.26; 0.15)	0.11
Partnered	-0.53 (-0.74; -0.34) ^§^	0.93	-0.24 (-0.47; -0.01) ^†^	0.44
**Depression**				
Single	-0.52 (-0.80; -0.24) ^§^	0.93	0.02 (-0.23; 0.27)	0.04
Partnered	-0.69 (-0.93; -0.44) ^§^	1.13	-0.34 (-0.61; -0.08) ^‡^	0.61
**Anxiety**				
Single	-0.62 (-0.98; -0.25) ^§^	0.86	-0.13 (-0.48; 0.23)	0.21
Partnered	-0.63 (-0.96; 0.30) ^§^	0.78	-0.15 (-0.49; 0.18)	0.22
**Post-trauma stress**				
Single	-0.36 (-0.67; 0.53) ^†^	0.55	-0.04 (-0.33; 0.34)	0.06
Partnered	-0.54 (-0.80; -0.28) ^§^	0.86	-0.38 (-0.68; -0.08) ^†^	0.55
**Dysfunction**				
Single	-0.36 (-0.68; -0.03) ^†^	0.62	-0.02 (-0.35; 0.39)	0.03
Partnered	-0.43 (-0.74; -0.13) ^‡^	0.77	-0.17 (-0.5; 0.16)	0.27

Mixed effect clustered models with multiple chained imputations. All the models were adjusted by age, gender, marital status, education, employment status, type of floor at home, type of health coverage, baseline TMHS, traumatic experiences, baseline assessment of sadness, baseline assessment of suffering, and time on study. City and LPCW were used as cluster variables. Models were also adjusted with the specific variables per scale described in Table 2 legend.

95% CI: 95% Confidence interval.

^†^ p<0.05.

^‡^ p<0.01.

^§^ p<0.001.

The between group effect size was measured with Cohen´s d (0.20–0.49: Small effect; 0.50–0.79: Moderate effect; ≥0.80: Large effect).

**Fig 4:**
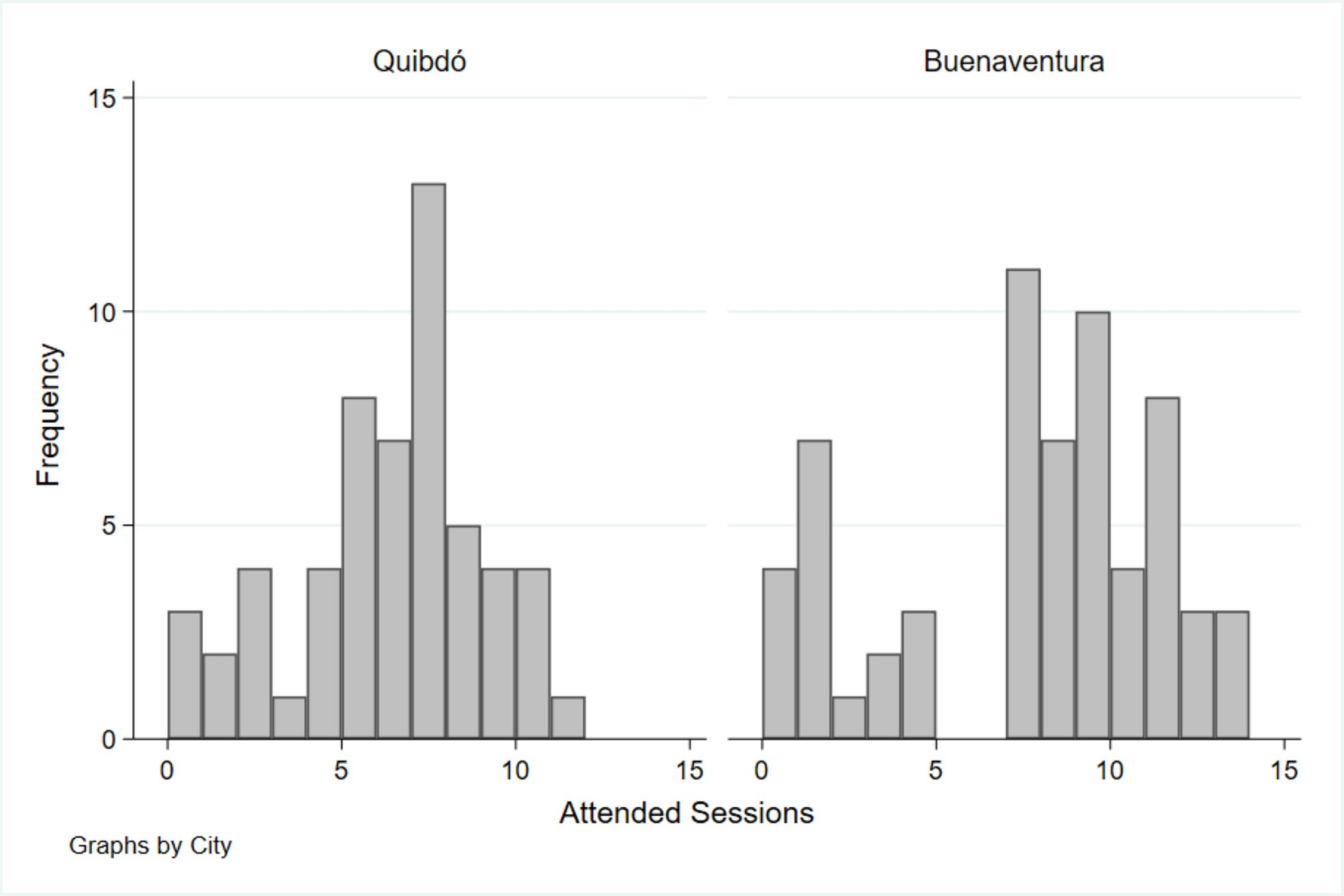
Histogram of number of participants’ CETA sessions by city. Only clients assigned to the CETA arm, with follow-up assessment, and with a LPCW assigned.

Number of sessions attended by participants was lower in Quibdó (mean 5.9, SD 2.8, median 6, interquartile range (iqr) 3) than in Buenaventura (mean 7.2, SD 3.9, median 8, iqr 6) (Two-sample Wilcoxon rank-sum, p = 0.006, Fig 4). There was not a significant difference in the number of sessions by LPCWs per city (Table 4).

**Table 4:** Median comparison of the number of participants’ sessions in CETA per lay psychosocial community workers (LPCW) and city.

Lay Psychosocial Community Workers	Greater than the median number of sessions		p-value*
	No	Yes	
**Buenaventura, n (%)**			0.20
11	7 (24.14)	6 (22.22)}	
12	4 (13.79)	1 (3.70)	
13	8 (27.59)	6 (22.22)	
14	8 (27.59)	6 (22.22)	
15	2 (6.90)	8 (29.63)	
**Quibdó, n (%)**			0.25
21	9 (25.71)	6 (21.43)	
22	13 (37.14)	5 (17.86)	
23	6 (17.14)	9 (32.14)	
28	7 (20.00)	8 (28.57)	

* Chi-square test

In the post-hoc analysis, the number of sessions were associated with lower TMHS, depression, anxiety, and PTSS only in Buenaventura, there were no significant changes based on the number of sessions in Quibdó (Table 5), although the clinical significance is low, given the range of the scales (0 to 3 points)

**Table 5:** Effect of the number of sessions in the mental health symptoms scales by city.

Scale/Sub-scale	Buenaventura β (95% CI)	p-value	Quibdó β (95% CI)	p-value
Total Mental Health Symptoms	-0.049 (-0.086; -0.012)	0.010	0.016 (-0.032; 0.064)	0.52
Depression	-0.052 (-0.092; -0.012)	0.011	0.030 (-0.022; 0.082)	0.26
Anxiety	-0.054 (-0.095; -0.013)	0.010	0.012 (-0.044; 0.067)	0.68
Post-trauma stress	-0.044 (-0.087; -0.0011)	0.044	0.0065 (-0.057; 0.070)	0.84
Dysfunction	-0.0064 (-0.037; 0.024)	0.68	-0.063 (-0.14; 0.014)	0.11

Mixed effect clustered models with multiple chained imputations. All the models were adjusted by age, gender, marital status, education, employment status, type of floor at home, type of health coverage, baseline TMHS, traumatic experiences, baseline assessment of sadness, baseline assessment of suffering, and time on study. LPCW was used as cluster variable. Models were also adjusted with the specific variables per scale described in Table 2 legend.

95% CI: 95% Confidence interval.

**Fig 5:**
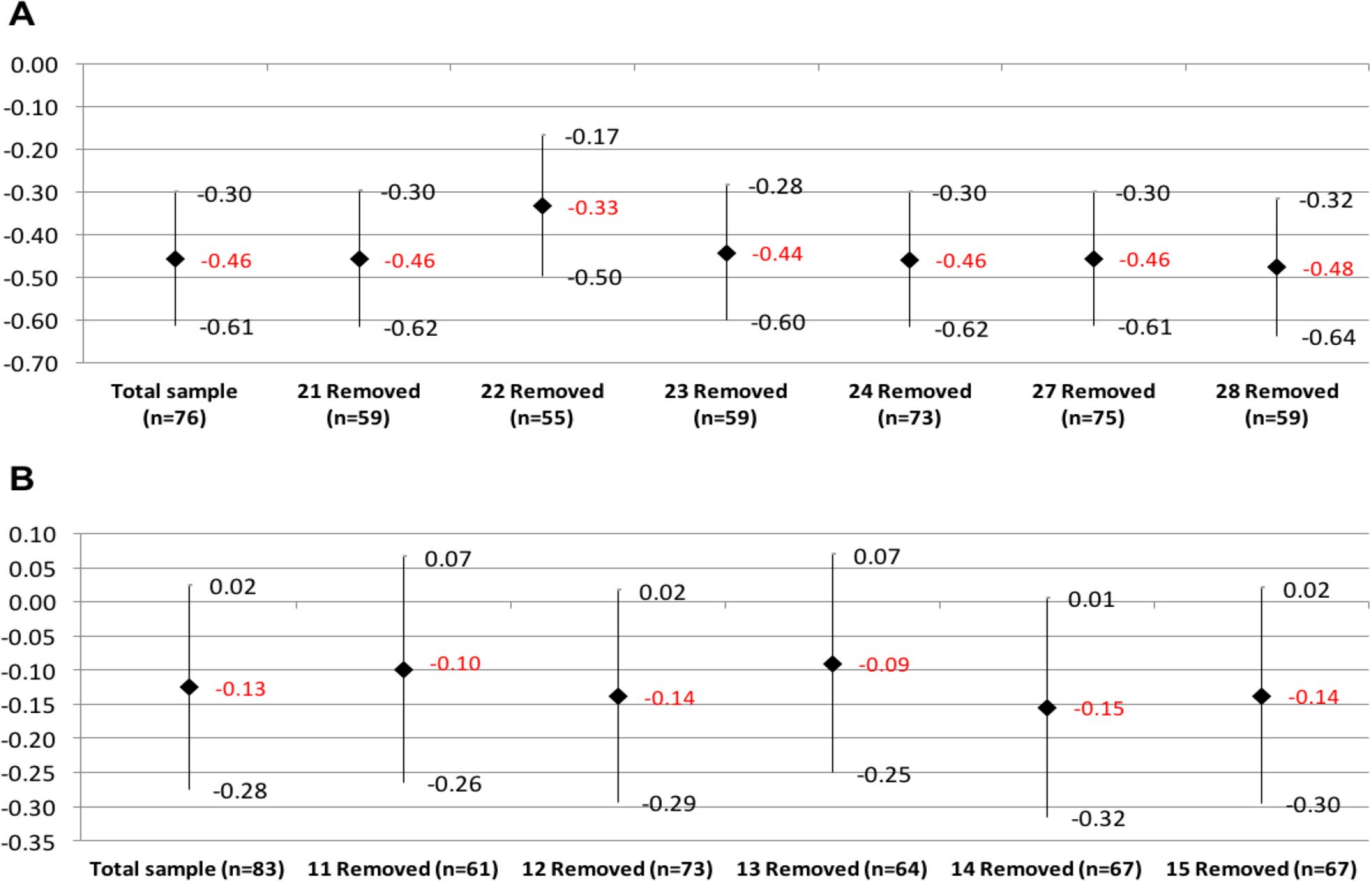
**Analysis of lay psychosocial community workers (LPCW) sensitivity:** Comparative of changes on the Total of Mental Health Symptoms (TMHS) scale and its 95% confidence intervals, as a result of the removal of a CETA counselor in (A) Buenaventura and (B) Quibdó. Data as shown is estimated out of the TMHS difference when comparing the effects of the intervention and the control group and its 95% confidence intervals controlled by age, gender, and marital status, clustered by LPCW and client.

Finally, sensitivity analyses of LPCWs effect on pooled TMHS scores were analyzed by city, removing each time a LPCW (Fig 5A and 5B). The LPCWs variable identified the assigned LPCW per every participant; in the case of Buenaventura it included six (6) LPCWs (#21, #22, #23, #24, #27, #28) plus an additional option (#98) for those respondents who did not have an assigned LPCW because they did not attend the first session of the intervention (16 clients). Two (2) LPCWs only attended 1 and 3 clients each; being the LPCWs #27 and #24, respectively. Both were suspended to provide attention in the program in the initial stages of the project implementation. The rest of LPCWs who saw clients during the entire project met an average of 18 clients with a range between 17 and 21 clients. Only the LPCW #22 attended 21 clients while the others attended 17 clients.

The 4 LPCWs from Buenaventura who participated during the whole project had significant reductions in clients’ TMHS, with percentages of change varying from -12.3% (LPCW #28) to -29.3% (LPCW #22). On the other hand, LPCWs who did not provide care beyond the start of the project had non-significant changes in the respondents of -13.7% and -17.7% (LPCWs #24 and #27, respectively). Those who did not have an assigned LPCW due to the loss of follow-up had a non-significant average increment in the symptoms of 2.3% (p = 0.68. Fig 5A and Table 6).

**Table 6:** Assessment of the effect of Lay psychosocial community workers (LPCWs) on the Total Mental Health Symptoms (TMHS) scale on CETA participants in Buenaventura and Quibdó.

Lay Psychosocial Community Workers	TMHS			
	Average Change^+^	IC 95%	% change^*^	p
**Buenaventura (n =**				
21	-0.46	-0.73; -0.18	-15.3%	0.001
22	-0.88	-1.13; -0.63	-29.3%	<0.0001
23	-0.52	-0.8; -0.25	-17.3%	<0.0001
24	-0.41	-1.04; 0.22	-13.7%	0.203
27	-0.53	-1.57; 0.5	-17.7%	0.315
28	-0.37	-0.65; -0.09	-12.3%	0.009
98	0.07	-0.25; 0.38	2.3%	0.681
**Quibdó**				
11	-0.2	-0.44; 0.05	-6.7%	0.122
12	-0.03	-0.39; 0.34	-1.0%	0.875
13	-0.24	-0.51; 0.02	-8.0%	0.076
14	-0.002	-0.28; 0.28	-0.1%	0.986
15	-0.07	-0.36; 0.22	-2.3%	0.626

^+^. Data shown are the estimated of TMHS’s change when the intervention is compared with the control group and the confidence intervals at 95% using mixed effect models controlled by age, genre, and marital status, clustered by LPCW.

^*^. Change of scale’s percentage:

In Quibdó, five (5) LPCWs were analyzed (#11, #12, #13, #14, #15). There were not respondents without an assigned LPCW. One of the LPCWs (#12) was pregnant during the intervention and was suspended from attending clients. The rest of the LPCWs attended from 16 to 22 clients. LPCWs #14 and #15 had 16 clients, LPCW #13 had 19 clients, and LPCW #11 had 22 clients. None of the LPCWs had a significant effect on clients’ symptoms; however, there was a non-significant symptom reduction (Fig 5B and Table 6).

In addition, a model was developed to evaluate the results of the LPCWs only with respondents who completed the treatment (adherence to protocol) and yet in that analysis no significant differences were found in respondents’ symptoms.

## Discussion

This trial demonstrated that CETA, a trans-diagnostic psychotherapy provided by lay community workers, was effective in decreasing TMHS, PTSS, depression and anxiety symptoms and dysfunction in one of the studied sites (Buenaventura) but was only effective in PTSS in the other (Quibdó). CETA had a moderate effect on PTSS among Afro-Colombian survivors in Buenaventura and a small effect in Quibdó. Effectiveness in Buenaventura was large for all other outcomes, excepting a moderate effect on dysfunction. In Quibdó, effects were small, and only effects in PTSS were significant. Individuals with partners showed moderate and large effects in Buenaventura and Quibdó after the therapy.[41]

Buenaventura’s findings are consistent with other trials,[20, 25, 31, 42, 43] showing how psycho-education, group therapy, behavioral and cognitive-behavioral therapy in low-income countries are effective for depression, anxiety and PTSS. CETA showed effectiveness under active armed-conflict settings in Buenaventura, similar to findings in a study among Congolese survivors of sexual violence led by lay community-workers,[40] but results in Quibdó and differences in results between two similar sites are not consistent with the literature.

The smaller effects of CETA in Quibdó show that results of an intervention delivered under similar conditions in two nearby, culturally, and ethnically alike sites can differ.[26, 44] In this trial, possible explanations include differences in the social context between the cities: while Quibdó has 89% of people living with unsatisfied basic needs, Buenaventura has significantly less with 36% of people living with unsatisfied basic needs, also, different armed actors are involved in each city. In Buenaventura, drug cartels and armed groups surrounding the drug business, while in Quibdó, the guerrilla warfare and armed groups based on illegal mining are the main sources of conflict and violence.[45–47]

Differences in the coping mechanisms and how urbanization and the conflict has changed the Afro-Colombian communities fabric could play also a role in the cities differences.[7] Buenaventura urbanization goes faster than Quibdó, mainly due to political and economic interests, and the differences in access to each city; e.g., Buenaventura is the main Colombian port on the Pacific ocean, therefore there are National efforts to improve accessibility to the city, while Quibdó is accessible mainly by plane due to the lack of investment in routes and the time that could take to access the city which could go from 6 hours to days. A better understanding of the cities differences in terms of violence, culture, and how they approach mental health could elucidate the differences in our results.

Variances in the delivery of the intervention may have played a role as well, as Quibdó had LPCWs with less community-work experience compared to Buenaventura. However, the sensitivity analysis of LPCWs did not show changes in the effect of the intervention in either city.

The analyses by LPCW, per city, have showed that there were not statistically significant differences of mean participants’ changing scores, among LPCWs, in the total mental health symptoms, neither in Quibdó nor in Buenaventura. This means that the treatment effects were homogenous among LPCWs per city. Nonetheless, CBI effects were significant for more scales and sub-scales in Buenaventura compared with Quibdó, and among significant results, the effects were better in Buenaventura than in Quibdó. Thus, as an attempt for understanding the differences of CBI effects between cities, several analyses, among CBI participants, were done taking into account the number of CBI sessions.

Additionally, dissimilar results might be partly due to a threshold effect in the number of sessions, since the mean number of sessions was lower in Quibdó compared to Buenaventura. A CETA trial with displaced Burmese adults exposed to violence showed that the mean session attendance was 9.7, similar to Buenaventura where CETA effects were larger.[20] Further analysis of the symptoms trends could help to understand the dose effect of the interventions.

### Limitations

A high proportion of participants were lost to follow-up. Possible causes include the ongoing violence in both places and difficulties in outreach in conflict-influenced displaced populations.[48] Nevertheless, our study had drop-out rates in line with other similar intervention studies in middle and low income settings, which range from 12% to 37%.[23, 40, 49]

This study does not include subsequent follow-ups to assess long-term effects of CETA. Literature has shown positive and sustainable results (at 6 months) for similar interventions,[40, 50] but future studies should include longer term follow up.

Despite efforts made by the team to achieve gender balance among groups, the majority of participants were women in both municipalities, thus, preplanned subgroup analysis by gender were not carried out. Difficulties when reaching men could be explained by the trend of reporting lower levels of mental distress,[51] their occupational activities, and the fact that a higher proportion of internally displaced men are killed or disappeared compared to woman. Finally, men could also fear that revealing their victim status might make them vulnerable to re-victimization by the armed groups.[48]

Given that violence and conflict remain active in the urban and rural settings where the volunteers resided, re-exposure to traumatic events might have occurred and the fact that participants were not evaluated for new traumatic experiences throughout the duration of the study could have an impact on the effectiveness of the intervention. We do not have enough evidence to tell if there were more violence incidents in one cite or the other, but in our experience, Buenaventura had more violence, e.g., neighborhoods of displaced people where block by illegal armed groups and became inaccessible for visitors at least three quarters of the trial.

### Conclusions

This intervention with community lay workers has the potential for being sustainable and accepted by their communities. This is important considering the limited number of mental health professionals present or available in these Colombian regions. Moreover, other intervention and implementation researches should be carried out in similar settings in Colombia, given the novel approach presented in this study.

This trial suggests that CETA can be effective in improving depression, anxiety, PTSS and function among victims of violence and torture in low-income and active conflict settings. Interestingly, the intervention proved to be much less effective in Quibdó, despite having the same researchers, methodology, trainers and supervision structure. It may be that assuming that a mental health intervention known to be effective in one situation will be effective in another, even in similar circumstances and population, is an unsafe assumption.[44]

More program-based research is recommended to broaden the understanding of what interventions are effective and under what circumstances. Policymakers should act on the available evidence to scale-up effective programs, but include outcome monitoring to ensure that expected impacts are actually achieved. In the case of Colombia, it is hoped that these research findings will contribute to the public dialogue on potential methodologies (e.g., PAPSIVI) available to the institutions implementing programs for victims of the conflict and torture.

## Supporting information

S1 File Validating an instrument for victims of violence in Colombia. (DOCX)

S2 File Research protocol–CETA trial—ACOPLE project. (PDF)

S3 File CONSORT 2010 checklist CETA trail. (PDF)

## References

[1] Internal Displacement Monitoring Centre—IDMC, Norwegian Refugee Council—NRC. Global Report on Internal Displacement GRID 2018. Geneva: IDMC, 2018 May 2018. Report No.

[2] Colombian Government, Unit for Victims. Official Victims' Registry Bogota D.C.: Colombian Government,; 2018 [updated Sep 1, 2018; cited 2018 Sept 13]. Available from: https://www.unidadvictimas.gov.co/es/registro-unico-de-victimas-ruv/37394.

[3] Albuja S, Arnaud E, Caterina M, Charron G, Foster F, Glatz AK, et al Global Overview 2014: People Internally Displaced by Conflict and Violence. Geneva: Internal Displacement Monitoring Centre. Norwegian Refugee Council; 2014 78 p.

[4] Franco Agudelo S. [Violence and Health in Colombia]. [Proceedings of the National Health Sector Forum Against Violence in Colombia: Policy and Plan of Action organized by the Ministry of Health and the Health and Development Corporation]. Bogota D.C: Ministry of Health; 1998 p. 27–39.

[5] Krug E, Dahlberg L, Mercy J, Zwi A, Lozano R. World Report on Violence and Health. Geneva: World Health Organization, 2002.

[6] Escobar A. Displacement, development, and modernity in the Colombian Pacific. Int Soc Sci J. 2003;55(175):157–67.

[7] Bonilla-Escobar FJ, Osorio-Cuellar GV, Pacichana-Quinayaz SG, Sanchez-Renteria G, Fandino-Losada A, Gutierrez MI. Do not forget culture when implementing mental health interventions for violence survivors. Cien Saude Colet. 2017;22(9):3053–9. Epub 2017/09/28. 10.1590/1413-81232017229.12982016 . 28954156 10.1590/1413-81232017229.12982016

[8] Osorio-Cuellar GV, Pacichana-Quinayaz SG, Bonilla-Escobar FJ, Fandino-Losada A, Gutierrez-Martinez MI. Perceptions about implementation of a Narrative Community-based Group Therapy for Afro-Colombians victims of Violence. Cien Saude Colet. 2017;22(9):3045–52. Epub 2017/09/28. 10.1590/1413-81232017229.00402016 . 28954155 10.1590/1413-81232017229.00402016

[9] Pacichana-Quinayaz SG, Osorio-Cuellar GV, Bonilla-Escobar FJ, Fandino-Losada A, Gutierrez-Martinez MI. Common Elements Treatment Approach based on a Cognitive Behavioral Intervention: implementation in the Colombian Pacific. Cien Saude Colet. 2016;21(6):1947–56. Epub 2016/06/09. 10.1590/1413-81232015216.07062015 . 27276543 10.1590/1413-81232015216.07062015

[10] Pérez-Olmos I, Fernández-Piñeres PE, Rodado-Fuentes S. [The prevalence of war-related post-traumatic stress disorder in children from Cundinamarca, Colombia]. Rev Salud Publica (Bogota). 2005;7(3):268–80. 16396416 10.1590/s0124-00642005000300003

[11] Sistiva-Castro DL, Sabatier C. [Socio-political violence, posttraumatic stress, and religion as a coping strategy. A comparative study in Colombia]. Revue francophone du stress et du trauma. 2005;5(2):97–107.

[12] Santaella-Tenorio J, Bonilla-Escobar FJ, Nieto-Gil L, Fandino-Losada A, Gutierrez-Martinez MI, Bass J, et al Mental Health and Psychosocial Problems and Needs of Violence Survivors in the Colombian Pacific Coast: A Qualitative Study in Buenaventura and Quibdo. Prehosp Disaster Med. 2018:1–8. Epub 2018/07/27. 10.1017/S1049023X18000523 . 30047356 10.1017/S1049023X18000523

[13] Pacichana-Quinayaz SG, Osorio-Cuellar GV, Gonzalez S, Bonilla-Escobar FJ, Gutierrez-Martinez MI. Relevance of Qualitative Research Approach in Evaluating Mental Health Interventions among Victims of Violence. Int J Med Students. 2015;3(3):170–1.

[14] Doctors Without Borders/Medicins Sans Frontieres (MSF). Colombia: An invisible problem. The psychological consequences of violence on ordinary people in the south of the country Bogota D.C: MSF; 2013 [updated Jul 8, 2013; cited 2015 Mar 10, 2015]. Available from: http://www.msf.org/article/colombia-invisible-problem.

[15] Comptroller General of the Republic, National Attorney General's Office, Office of the Ombudsman, National Board of Victim Participation. First report to the Congress of the Republic 2013–2014. Commission for Monitoring and Compliance Monitoring of Act 1448 of 2011. Bogota D.C.2014. p. 612.

[16] Presidential Agency for Social Action and International Cooperation (Accion Social). [Register of Displaced Population]. Bogotá D.C: Presidential Agency for Social Action and International Cooperation, 2009.

[17] Calvani S, Dupuy PC, Liller S. Violence, Crime and Illegal Arms Trafficking in Colombia. Bogota D.C: United Nations Office on Drugs and Crime; 2006 [cited 2015 Mar 15]. Available from: http://www.unodc.org/pdf/Colombia_Dec06_en.pdf.

[18] Murray LK, Dorsey S, Haroz E, Lee C, Alsiary MM, Haydary A, et al A Common Elements Treatment Approach for Adult Mental Health Problems in Low- and Middle-Income Countries. Cogn Behav Pract. 2014;21(2):111–23. Epub 2015/01/27. 10.1016/j.cbpra.2013.06.005 ; PubMed Central PMCID: PMCPMC4304666. 25620867 10.1016/j.cbpra.2013.06.005 PMC4304666

[19] Weiss WM, Murray LK, Zangana GA, Mahmooth Z, Kaysen D, Dorsey S, et al Community-based mental health treatments for survivors of torture and militant attacks in Southern Iraq: a randomized control trial. BMC Psychiatry. 2015;15:249 Epub 2015/10/16. 10.1186/s12888-015-0622-7 ; PubMed Central PMCID: PMCPMC4605204. 26467303 10.1186/s12888-015-0622-7 PMC4605204

[20] Bolton P, Lee C, Haroz EE, Murray L, Dorsey S, Robinson C, et al A transdiagnostic community-based mental health treatment for comorbid disorders: development and outcomes of a randomized controlled trial among Burmese refugees in Thailand. PLoS Med. 2014;11(11):e1001757 10.1371/journal.pmed.1001757 ; PubMed Central PMCID: PMCPMC4227644. 25386945 10.1371/journal.pmed.1001757 PMC4227644

[21] Resick PA, Nishith P, Weaver TL, Astin MC, Feuer CA. A comparison of cognitive-processing therapy with prolonged exposure and a waiting condition for the treatment of chronic posttraumatic stress disorder in female rape victims. J Consult Clin Psychol. 2002;70(4):867–79. Epub 2002/08/17. ; PubMed Central PMCID: PMC2977927. 12182270 10.1037//0022-006x.70.4.867 PMC2977927

[22] Resick PA, Williams LF, Suvak MK, Monson CM, Gradus JL. Long-term outcomes of cognitive-behavioral treatments for posttraumatic stress disorder among female rape survivors. J Consult Clin Psychol. 2012;80(2):201–10. Epub 2011/12/21. 10.1037/a0026602 ; PubMed Central PMCID: PMC3336190. 22182261 10.1037/a0026602 PMC3336190

[23] Bolton P, Bass J, Neugebauer R, Verdeli H, Clougherty KF, Wickramaratne P, et al Group interpersonal psychotherapy for depression in rural Uganda: a randomized controlled trial. Jama. 2003;289(23):3117–24. 10.1001/jama.289.23.3117 12813117 10.1001/jama.289.23.3117

[24] World Health Organization, World Organization of National Colleges Academies, Academic Associations of General Practitioners/Family Physicians. Integrating mental health into primary care: a global perspective. Geneva: World Health Organization; 2008.

[25] Patel V, Simon G, Chowdhary N, Kaaya S, Araya R. Packages of care for depression in low-and middle-income countries. PLoS Med. 2009;6(10):e1000159 10.1371/journal.pmed.1000159 19806179 10.1371/journal.pmed.1000159 PMC2747016

[26] Murray LK, Tol W, Jordans M, Sabir G, Amin AM, Bolton P, et al Dissemination and implementation of evidence based, mental health interventions in post conflict, low resource settings. Intervention. 2014;12:94–112. 10.1097/WTF.0000000000000070 28316559 10.1097/WTF.0000000000000070 PMC5356225

[27] Heartland Alliance International. Treating survivors in Colombia. Chicago: Heartland Alliance International, 2011.

[28] Derogatis LR, Lipman RS, Rickels K, Uhlenhuth EH, Covi L. The Hopkins Symptom Checklist (HSCL): a self-report symptom inventory. Behav Sci. 1974;19(1):1–15. 4808738 10.1002/bs.3830190102

[29] Mollica RF, Caspi-Yavin Y, Bollini P, Truong T, Tor S, Lavelle J. The Harvard Trauma Questionnaire. Validating a cross-cultural instrument for measuring torture, trauma, and posttraumatic stress disorder in Indochinese refugees. J Nerv Ment Dis. 1992;180(2):111–6. Epub 1992/02/01. . 1737972

[30] Weathers FW, Litz BT, Herman D, Huska J, Keane T. The PTSD checklist-civilian version (PCL-C). Boston, MA: National Center for PTSD 1994.

[31] Weisz JR, Chorpita BF, Palinkas LA, Schoenwald SK, Miranda J, Bearman SK, et al Testing standard and modular designs for psychotherapy treating depression, anxiety, and conduct problems in youth: A randomized effectiveness trial. Arch Gen Psychiatry. 2012;69(3):274–82. 10.1001/archgenpsychiatry.2011.147 22065252 10.1001/archgenpsychiatry.2011.147

[32] Chorpita BF, Daleiden EL. Mapping evidence-based treatments for children and adolescents: application of the distillation and matching model to 615 treatments from 322 randomized trials. J Consult Clin Psychol. 2009;77(3):566 10.1037/a0014565 19485596 10.1037/a0014565

[33] Barlow DH, Farchione TJ, Fairholme CP, Ellard KK, Boisseau CL, Allen LB, et al Unified protocol for transdiagnostic treatment of emotional disorders: Therapist guide. United Kingdom: Oxford University Press; 2010.

[34] Murray L, Dorsey S. Intervention: Colombian version. Therapist manual. Colombia: Heartland Allaince International; 2011.

[35] Congress of Colombia. Act 1090 2006 (Wednesday September 6) whereby the exercise of the profession of psychology is regulated, dictates the Code of Ethics and Bioethics and other provisions. 46.383 ed. Bogota D.C.: Congress of Colombia; 2006.

[36] Tsuang MT, Tohen M, Jones P. Textbook of Psychiatric Epidemiology. New Jersey: John Wiley & Sons, Inc; 2011 664 p.

[37] Horn EE, Xu Y, Beam CR, Turkheimer E, Emery RE. Accounting for the physical and mental health benefits of entry into marriage: a genetically informed study of selection and causation. J Fam Psychol. 2013;27(1):30–41. Epub 2012/10/24. 10.1037/a0029803 ; PubMed Central PMCID: PMC3645280. 23088795 10.1037/a0029803 PMC3645280

[38] Fandiño-Losada A, Forsell Y, Lundberg I. Demands, skill discretion, decision authority and social climate at work as determinants of major depression in a 3-year follow-up study. International archives of occupational and environmental health. 2013;86(5):591–605. 10.1007/s00420-012-0791-3 22760975 10.1007/s00420-012-0791-3

[39] Cohen J. Statistical Power Analysis for the Behavioral Sciences: L. Erlbaum Associates; 1988.

[40] Bass JK, Annan J, McIvor Murray S, Kaysen D, Griffiths S, Cetinoglu T, et al Controlled trial of psychotherapy for Congolese survivors of sexual violence. The New England journal of medicine. 2013;368(23):2182–91. Epub 2013/06/07. 10.1056/NEJMoa1211853 . 23738545 10.1056/NEJMoa1211853

[41] Holt-Lunstad J, Birmingham W, Jones BQ. Is there something unique about marriage? The relative impact of marital status, relationship quality, and network social support on ambulatory blood pressure and mental health. Annals of behavioral medicine: a publication of the Society of Behavioral Medicine. 2008;35(2):239–44. Epub 2008/03/19. 10.1007/s12160-008-9018-y . 18347896 10.1007/s12160-008-9018-y

[42] Olatunji BO, Cisler JM, Deacon BJ. Efficacy of cognitive behavioral therapy for anxiety disorders: a review of meta-analytic findings. Psychiatr Clin North Am. 2010;33(3):557–77. 10.1016/j.psc.2010.04.002 20599133 10.1016/j.psc.2010.04.002

[43] Ekers D, Richards D, Gilbody S. A meta-analysis of randomized trials of behavioural treatment of depression. Psychol Med. 2008;38(05):611–23. 17903337 10.1017/S0033291707001614

[44] Hohmann AA, Shear MK. Community-based intervention research: coping with the "noise" of real life in study design. The American journal of psychiatry. 2002;159(2):201–7. Epub 2002/02/02. 10.1176/appi.ajp.159.2.201 . 11823259 10.1176/appi.ajp.159.2.201

[45] Washington Office on Latin America (WOLA). Hostages in Our Own Territories: Afro-Colombian Rights under Siege in Chocó. Washington D.C.: WOLA, 2012 Mar 28, 2012. Report No.

[46] Carrillo A. Forced Displacement and Gender-based Sexual Violence Buenaventura, Colombia: Brutal realities. Bogota D.C: NRC; 2014 [cited 2015 Mar 10]. Available from: http://www.nrc.no/arch/_img/9183706.pdf.

[47] Administrative Department of National Statistics (DANE). [Colombia. Unsatisfied Basic Needs—UBN, for total by city and country]. Bogotá D.C: Colombian Government; 2012.

[48] Shultz JM, Muñoz García N, Gómez Ceballos ÁM, Hernandez Florez LJ, Araya R, Verdeli H, et al Outreach to internally displaced persons in Bogotá, Colombia: challenges and potential solutions. Disaster Health. 2014;2(1):1–6. 10.4161/dish.29249 28229001 10.4161/21665044.2014.954500 PMC5314910

[49] Patel V, Weiss HA, Chowdhary N, Naik S, Pednekar S, Chatterjee S, et al Effectiveness of an intervention led by lay health counsellors for depressive and anxiety disorders in primary care in Goa, India (MANAS): a cluster randomised controlled trial. Lancet. 2010;376(9758):2086–95. 10.1016/S0140-6736(10)61508-5 21159375 10.1016/S0140-6736(10)61508-5 PMC4964905

[50] Cloitre M, Stovall-McClough KC, Nooner K, Zorbas P, Cherry S, Jackson CL, et al Treatment for PTSD related to childhood abuse: a randomized controlled trial. The American journal of psychiatry. 2010;167(8):915–24. Epub 2010/07/03. 10.1176/appi.ajp.2010.09081247 . 20595411 10.1176/appi.ajp.2010.09081247

[51] Koopmans GT, Lamers LM. Gender and health care utilization: the role of mental distress and help-seeking propensity. Soc Sci Med. 2007;64(6):1216–30. Epub 2006/12/30. 10.1016/j.socscimed.2006.11.018 . 17194514 10.1016/j.socscimed.2006.11.018

