# Supplementary material for: A randomized controlled trial of a transdiagnostic cognitive-behavioral intervention for Afro-descendants’ survivors of systemic violence in Colombia
Source: PLoS One. 2018 Dec 10;13(12):e0208483. doi: 10.1371/journal.pone.0208483 (PMC6287825; doi:10.1371/journal.pone.0208483)
Supplement: S1 File — (DOCX) [file pone.0208483.s001.docx]

**S1 File. Validating an instrument for victims of violence in Colombia.**

We conducted a validation study with a sample of Afro-Colombians from the two Colombian cities, Buenaventura and Quibdó. It was carried out in 2011 to evaluate phychometric properties of a culturally adapted mental health and dysfunction assessment tool based on the Hopkins Symptom Checklist (HSCL-25), [[28](#_ENREF_28)] the Harvard Trauma Questionnaire (HTQ), [[29](#_ENREF_29)] PTSD CheckList – Civilian Version (PCL-C),[[30](#_ENREF_30)] and a qualitative study to identify mental health symptoms of Afro-Colombian victims of violence.[[12](#_ENREF_12)] The questionnaire also included socio-demographic, socioeconomic, traumatic experiences, access to health care services, and support networks variables.

To recruit participants for the validation study, we first contacted key informants (KI) and asked them to share with us the names of individuals from their community who they knew suffered from the problems previously identified as the most common problems of individuals suffering from violence and torture in these communities (approximately 50 participants per problem). We also asked KI to provide the names of 50 participants who they know had none of these problems. A total of 197 and 202 individuals from Quibdó and Buenaventura, respectively, participated in the validation study. Following the information provided by KI, in Quibdó we interviewed a total of 49 individuals with no problems, 42 suffering from *tristeza* (sadness), 45 from *sufrimiento* (suffering), and 47 from *trauma psicológico* (psychologic trauma). A person could have multiple problems so there was could overlap of individuals across problems. In Buenaventura, we interviewed 45 individuals with no problems, 52 suffering from *tristeza*, 63 from *sufrimiento*, and 67 from *trauma psicológico*. All interviewers were blinded to the initial status classification when the lists were made.

The criterion validity testing for scales was performed based on: a) classification of participants as indicated by KI; b) classification of participants based on participants’ responses on whether they had or not these problems; c) only participants for whom we had records of being originally concordant (e.g., the KI believed they had one of these problems, and the participant agreed with this determination; concordance between KI and participants in Quibdó and Buenaventura was 80.95% and 82.69% for *tristeza*, respectively, 82.22% and 79.37% for *sufrimiento*, and 44.68% and 46.27% for *trauma psicológico*.

Results from criterion analyses in the Quibdó sample showed that individuals who were identified by key informants as having *tristeza*, *sufrimiento* and *trauma psicológico* had on average significantly higher scores in all scales, including HSCL Depression scale, HSCL Anxiety scale, PCL-C scale, General Symptoms scale, and Qualitative scale, compared with those without problems (independent group t-tests, p-value<0.05). We found very similar results when using the classification based on participants’ responses and using only participants for whom we had records of being concordant. The only different finding was that when only using participants with concordant records, the group with *trauma psicológico* had a significantly higher mean score in the function scale compared to the group without problems (1.22 vs. 0.88, respectively; p-value<0.05).

Results from criterion analyses in the Buenaventura sample showed that when using the classification of participants as indicated by KI, there were only significant differences in mean scores in the Anxiety scale (when those with *sufrimiento* and *trauma psicológico* were compared to those without problems). However, when using the classification of participants based on participants’ responses, we found that individuals having problems had significantly higher mean scores in all scales of the instrument when compared to those without problems. This was also the case when only using participants with concordant records (one exception was found when comparing those with *tristeza* vs. those without problems: score in the function scale was only marginal, p-value=0.051).

**Internal consistency reliability:**

In both municipalities the instrument showed high internal consistency (Cronbach’s alpha was 0.955 in Quibdó and 0.965 in Buenaventura). The internal consistency was also high for most scales, as shown in table S1, indicating that items assessing the same underlying concept agreed with each other. Further item analyses did not support the removal of any of the items from any of the scales (no improvement of alpha after items removal).

Table S1. Internal Consistency (Cronbach’s Alpha)

| **Scales** | **Quibdó (n=197)** | **Buenaventura (n=202)** |
| --- | --- | --- |
| HSCL depression section score | 0.84 | 0.85 |
| HSCL anxiety section score | 0.88 | 0.88 |
| PCL-C scale | 0.85 | 0.89 |
| General Symptoms scale | 0.72 | 0.74 |
| Qualitative scale | 0.85 | 0.87 |
