# Supplementary material for: A randomized controlled trial of a transdiagnostic cognitive-behavioral intervention for Afro-descendants’ survivors of systemic violence in Colombia
Source: PLoS One. 2018 Dec 10;13(12):e0208483. doi: 10.1371/journal.pone.0208483 (PMC6287825; doi:10.1371/journal.pone.0208483)
Supplement: S2 File — (PDF) [file pone.0208483.s002.pdf]

Study Protocol:

EVALUATION OF TWO COMMUNITY-BASED MENTAL HEALTH INTERVENTIONS FOR AFRO-COLOMBIANS VICTIMS OF VIOLENCE

Francisco J. Bonilla-Escobar, MD, MSc; Andrés Fandiño-Losada, MD, MSc, PhD; Diana M. Martínez-Buitrago, MD, MSc; Julián Santaella-Tenorio, MV, MSc; Daniel Tobón-García, MD, Edgar J. Muñoz-Morales, MD, MSc; María I. Gutiérrez-Martínez, PhD.

Institute for Peace Promotion and Injury/Violence Prevention (CISALVA), Faculty of Health, Universidad del Valle, Cali, Colombia

Judith K. Bass, PhD; Laura K. Murray, PhD; Paul Bolton MB BS.

Department of International Health and Department of Mental Health, Bloomberg School of Public Health, Johns Hopkins University, Baltimore, MD, USA

Shannon Dorsey, PhD.

Department of Psychology, University of Washington. Seattle, WA, USA.

Heartland Alliance International, Cali, Colombia and Chicago, IL, USA

This study is made possible by the generous support of the American people through the United States Agency for International Development (USAID), contract number AID-OAA-A-10-00046. The contents are the responsibility of the Cisalva Institute of Universidad del Valle and Heartland Alliance International and do not necessarily reflect the views of USAID or the United States Government.

Cali, September 2011

Universidad del Valle

TABLE OF CONTENTS

List of Tables 4
ABSTRACT. 5
1. INTRODUCTION 7
2. THOERETICAL FRAMEWORK 11
2.1. Violence and its Mental Health Consequences 11
2.2. Treatment Models for Victims of Violence 12
3. OBJECTIVES 16
3.1. General Objective 16
3.2. Specific Objectives 16
4. METHODOLOGY 17
4.1. Phase 1: Qualitative Study of the Effects of Violence 17
4.2. Phase 2: Design and Validation of the Instrument Based on Qualitative Data of Victims of Violence and Torture 18
4.3. Phase 3: Design and Pilot Test of Mental Health Intervention 20
4.3.1. Selection and Adaptation of Interventions 20
4.3.2. Pilot Test of Interventions and Fieldwork 21
4.4. Phase 4. Randomized Controlled Trial. Implementation and Evaluation of the Mental Health Interventions 22
4.4.1. Trial Design 22
4.4.2. Participants 22
4.4.3. Selection Criteria 22
4.4.3.1. Inclusion Criteria 22
4.4.3.2. Exclusion Criteria 23
4.4.4. Description of Interventions 23
4.4.4.1. Common Elements Treatment Approach (CETA) 23
4.4.4.2. Narrative Community-Based Group Therapy, NCGT 24
4.4.4.3. Wait-list Control 26
4.4.5. Enrollment Method and follow up assessment 26
4.4.6. Training of Participants in the research 27
4.4.6.1. Lay Psychosocial Community Workers (LPCW) 27
4.4.6.2. Psychologists 27
4.4.6.3. Interviewers 27

4.4.7. Monitoring and Supervision of the Intervention 28
4.4.7.1. Administrative Monitoring of the Process: 28
4.4.7.2. Monitoring of the Intervention Process 28
4.4.8. Definitions of Loss to Follow-up 31
4.4.9. Primary and secondary outcomes 32
4.4.10. Instrument 32
4.4.11. Sample Size 33
4.4.12. Randomization 35
4.4.13. Evaluation of the Psychosocial Interventions 35
4.4.13.1. Quantitative Impact of the Intervention 35
4.4.13.2. Qualitative Impact of the Intervention 37
4.4.14. Statistical Analysis Plan. 37
4.4.14.1. Multiple imputation 38
4.4.14.2. Selection of covariates for final mixed models 38
4.4.14.3. ITT Final Mixed Models 39
4.4.14.4. Subgroups analyses 39
4.4.14.5. Sensitivity Analyses with Follow-up Predictors 39
4.4.14.6. Sensitivity Analyses excluding regression outliers 40
4.4.14.7. Effect Sizes 40
4.5. Ethical considerations 40
5. TIMETABLE 43
6. REFERENCES. 46
6.1. Appendix No 1. Survey 50
6.2. Appendix No 2. Informed Consent 59
6.3. Appendix No 3. Manual of Common Elements Treatment Approach (CETA) 59
6.4. Appendix No 4. Manual of Narrative Community-Based Group Therapy (NCGT) 59
6.5. Appendix No 5. Participant Referral Form for Adverse Events 59
6.6. Appendix No 6. Security Protocol Manual 59

List of Tables

Table 1. Estimate of the displaced population in Buenaventura, years 2007-2008..... 8
Table 2. Distribution of study participants by intervention group and capacity of participants per Lay Psychosocial Community Workers (LPCW) in each city. 34

ABSTRACT.

Background: There are mental health consequences for individuals and communities that have been exposed to violence. The armed conflict in Colombia has caused death and massive displacement. In the Pacific region, displacement has been preponderant in Buenaventura and Quibdó. Evidence regarding effectiveness of mental health interventions is lacking in low-income settings, especially in areas with active conflict.

Objective: To evaluate two community interventions in mental health designed to decrease symptoms of depression, anxiety, and post-traumatic stress symptoms in Afro-Colombian victims of violence in Buenaventura and Quibdó.

Methods: A single-blind randomized controlled trial will be conducted in two municipalities of the Colombian Pacific in which participants will be randomly assigned to a cognitive-behavioral intervention -Common Elements Treatment Approach (CETA) (Group A), a Narrative Community-Based Group Therapy (NCGT) Intervention (Group B) and a control/waiting list group. Assessments of mental health symptoms and dysfunction will be performed at baseline and two weeks after the treatment for Group A and B participants and after three to four months in the control group. Symptoms will be assessed with a validated survey which includes elements from the Hopkins Symptom Checklist, the Harvard Trauma Questionnaire and relevant local symptoms; dysfunction will be assessed with a locally-validated gender-specific scale. Missing values will be handled using multiple imputation methods. Means will be calculated for scales and sub-scales. The primary outcomes will be changes in depression, anxiety and post-traumatic stress symptoms; secondary outcomes will be changes in the Total Mental Health Symptom (TMHS) and dysfunction scales. Differences at baseline will be assessed with the specific test accordingly to the type of variable. Using mixed models we will perform intention to treat analyzes and sensitivity analyzes removing those participants without follow up. Co-variables included in mixed-models will be those significant at the $p<0.10$ level identified using: 1) simple logistic regressions clustered by counselor to identify baseline differences between intervention and control groups; 2) mixed models to determine interactions between potential co-variables and time on symptoms and dysfunction scores. Furthermore, models will be adjusted for age, gender, education and marital status. Sub-groups analyzed by gender and civil status will be conducted. Extreme values in scales (> or < 4 standard deviations) will be identified as potential outliers and will be excluded in a sensitivity analysis to evaluate its impact on intervention outcomes. Effect sizes will be calculated using Cohen’s d statistic

Expected results: Evaluation of the effectiveness of two mental health interventions in order to describe alternatives for treatment in Afro-Colombian victims of violence in the Colombian Pacific Coast.

Keywords: Depression; Anxiety; Stress Disorders, Post-Traumatic; Cognitive Therapy; Narrative Therapy; Violence; Torture; Mental Health; Community Mental Health Services.

1. INTRODUCTION

The present study is framed within the Program called "Community-Based Treatment Services for Afro-Colombian Victims of Conflict and Torture (ACOPLE)", developed by Heartland Alliance International (HAI), Association of Displaced African Descendants (AFRODES), and CISALVA Institute at the Universidad del Valle in Colombia, in conjunction with the Secretaries of Health of the municipalities of Quibdó and Buenaventura, with technical assistance from members of the Department of International Health and Department of Mental Health, Bloomberg School of Public Health of John Hopkins University (JHU),[1] registered at *ClinicalTrials.gov* with code NCT01856673 (http://clinicaltrials.gov/ct2/show/NCT01856673).

The Afro-Colombian communities in the Pacific region of the country have been the target of ongoing attacks for the past several decades. As a consequence of this situation and poor socio-economic conditions, there is limited adequate care and accompaniment for the survivors of the conflict, especially if they need to recover societal bonds and functionality. This project seeks to identify an effective treatment to reduce the mental health symptoms generated by violence, while promoting mechanisms to generate sustainable mental health services in these regions.

In order to propose solutions that are feasible and effective for the community in terms of mental health treatment, it is necessary to first understand the socio-cultural context of the affected population and the dynamics of violence that, for years, has affected this population. Understanding these contexts allows for the cultural and language-appropriate adaptation of treatment alternatives that have been implemented in other settings. The incorporation of context-specific aspects into interventions reduces the potential of adverse events in the community.

The ongoing armed conflict in Colombia has generated multiple confrontations primarily in rural communities, causing the death of thousands and the displacement of large masses to nearby major urban areas. On the Pacific coast, a region where many armed groups compete for resources in the area, violence has intensified, which has generated an acute increase in Afro-Colombian internal displacement from the rural areas to the two most important towns in the region: Buenaventura in Valle del Cauca and Quibdó in Chocó.

The armed conflict in the pacific zone includes the presence of the Armed Forces of Colombia, paramilitary groups, the guerrilla groups (Revolutionary Armed Forces of Colombia (FARC) and National Liberation Army (ELN)) and emerging criminal groups dedicated to drug trafficking (BACRIM). These BACRIM have violated the human rights of people living in this area, carried out several massacres, generated massive displacements and conducted torture through the systematic elimination of community leaders.

Buenaventura is a vital municipality for the country's economy and is now the poorest municipality of Valle del Cauca. The percentage of the population with basic needs unmet is three times that found in Cali (the capital city of Valle del Cauca). Basic services are well below the departmental average and literacy rates, which are comparable to those found in Chocó (the Department were Quibdó is located), are low.[2] The internal armed conflict taking place in the country has had serious consequences in Buenaventura, where violence has raged since 1998. Buenaventura is becoming a strategic corridor for drug output to various countries, is increasing illicit crop growth in rivers, and the river mouths have become storage sites for drugs, supplies and fuel.[3] Some displacement figures are presented below.

Table 1. *Estimate of the displaced population in Buenaventura, years 2007-2008*

**Displaced Population**	**2007**	**2008**
Arriving persons	8,921	3,580
New homes	1,778	686
Expelled persons	12,322	5,769
Expelled homes	2,780	1,329
Expelled women	6,709	3,127
Expelled men	5,613	2,642
Arriving women	4,892	1,995
Arriving men	4,029	1.585
Expelled minors	5,902	2,769
Arriving minors	4,438	1,799

Source: Presidential Agency for Social Action and International Cooperation.[4]

Quibdó, capital of the municipality of Chocó, is the only center located between the mountains and the coast. It is at the center of a natural link that exists between the two Colombian coastlines, via the Atrato and San Juan rivers. The relationship of centrality

converges in the San Pablo isthmus area, specifically between Quibdó and Istmina, and is explained by the fluvial communication between the two great Atrato and San Juan rivers. The phenomenon of displacement to urban areas and internal mobilization among districts has been intensified by violence generated by armed conflict forces. This has created concentrated displacement centers in cities such as Tutunendo, La Troje, Negua, Villa del Rosario, Boca de Naurita and Quibdó, which are now the largest recipients of these populations.[5,6]

Furthermore, as the capital of the department, Quibdó currently has 4,014 displaced people: 983 men, 1,189 women and 1,843 children (defined as under 18 years of age), about 400 households.[7] A large percentage comes from the Lower and Middle Atrato, Juradó, Urabá, Bagadó, Negua, and El Dieciocho. The areas of urban settlement are located in the neighborhoods of Villa España, Coliseo and Cascorba, which receive comprehensive care from the Municipality Social Solidarity Network.

In 2002, there was over 4,000 politically motivated murders in the Pacific Regionin combined with more than 1,000 people reported missing at least 2,700 people kidnapped.. Violence in the region is due to the resurgence of paramilitary groups and drug traffickers who are financed through the forced seizure of arable land, mineral resources and drugs that are taken from southern Colombia to the Pacific coast for shipment to other countries.[7] It is estimated that 90% of forced disappearances and 55% of the acts of torture reported in the area are committed by paramilitary groups.[7]

Between July 2003 and June 2008 there was a total of 899 reports or torturein the area, of which 86.3% of torture victims were men while, 168 victims reported psychological abuse.[8]. These figures additionally are vastly minimized due to the majority of cases of torture in the region going unreported. The magnitude of the problem has not been fully determined and it is possible that a large number of people still continue to suffer from violence and torture in silence.[6]

The 2009 report from the Colombian Government Social Action program reports that 90% of the displaced population that has reached Buenaventura and Quibdó is Afro-Colombian (a total of 67,795 displaced persons in Buenaventura and 42,267 in Quibdó).[9]

Despite this, few mental health services are readily available for survivors of the conflict. The importance and need for these services only gained some recognition and attention from the State after the Victims Law (1448) was passed in 2011. Public health programs targeting this population are still in their infancy and are showing definite strategic and methodological weaknesses.

In 2009 the organization Heartland Alliance International (HAI) began developments to determine the mental health needs of Afro-Colombians in Colombia's Pacific coast, a process that also included the identification of institutions interested in the subject of providing appropriate treatment and solutions to the problems of victims due to violence in this region. In this initial phase, HAI identified that the availability of mental health help for the population living in Buenaventura and Quibdó is limited. Noting that there was a single specialist in Buenaventura and none in Quibdó, which shows a lack of trained personnel to meet the needs of individuals traumatized by violence.[10] These services, along with the research study of two mental health interventions constitute the need for ACOPLE.

2. THOERETICAL FRAMEWORK

2.1. Violence and its Mental Health Consequences

The long-term consequences of violence on health is well documented in the surrounding literature and evidence concludes that it can affect the physical , psychological and social recovery of victims.[11]

Violence is defined by the UN Convention Against Violence and the Criminal Code of the United States as "any act by which severe pain or suffering, whether physical or mental, is intentionally inflicted on a person for such purposes as obtaining from him or her or a third person information or a confession, punishing him or her for an act he or she or a third person has committed or is suspected of having committed, or intimidating or coercing him or her or a third person, or for any reason based on discrimination of any kind, when such pain or suffering is inflicted by or at the instigation of or with the consent or acquiescence of a public official or other person acting in an official capacity".[11]

The consequences of violence on the mental health of individuals is referred to as Post Traumatic Stress Disorder (PTSD). The most common symptoms associated with PTSD include, but are not limited to: flashbacks, escapism, agitation, feelings of guilt (that manifest in various ways such as memory problems and concentration), sleep problems, nightmares, hypertension and anxiety when confronted with reminders of the trauma, intrusive thoughts and memories of traumatic events, increased irritability and difficulties with interpersonal relationships, depression. isolation, anxiety and other somatic problems.[13](Is this whole list necessary?)

The effects of violence cause severe discomfort in survivors, affecting their quality of life and ability to function in daily life. Symptoms such as chronic pain, problems with interpersonal relationships and depression prevent the individual from performing basic tasks at home, school, work, and at the community. The degree of dysfunction is related to several factors, including the severity and duration of the traumatic event, the existence of family and community support, and individual variations in terms of susceptibility to depression.[14] Violence has the unique ability to isolate people, away from both their families and their social networks. The resulting suppression and PTSD limit social interaction of the victims and creates fear in the survivors as well as people who surround them.[11] Thus, the effects of

violence and trauma on individuals also have an impact on family, community and society functioning properly.

.[11]It is curious considering the rise of reported victims of violence that there is still no reliable and comprehensive treatment plan or system of services available for this growing population in need.

Given the interrelated dimensions of health from the physical, mental and social aspects, it is crucial that victims of violence are holistically met when an option in health care is provided;;[15] when one dimension is neglected the effects of violence become deeper.[16]

The victims of displacement are among the most vulnerable populations worldwide and unlike refugees have not yet crossed a border that would allow them a reprieve from conflict. In 2007, 1% of the global population was considered displaced due to armed conflict, violence, development projects and large-scale natural disasters.[17] In 2011 Colombia, with more than 5.7 million Internally Displaced Persons, is in the first place for the greatest total displaced population and currentlyhas the second largest number of persons displaced due to violence after Syria.[18]

In Colombia in 2002 a study , documented the existence of PTSD in children, youth and adults exposed to war and sociopolitical violence.[19-22] Researchers found that multiple exposures to traumatic events of violence, in conjunction with social conditions of poverty, displacement, racial or ethnic discrimination, resulted in severe traumatic anguish and complexes.. Health factors were multidimensional and affected the functionality of the individual, family and community.[19,21,22]

2.2. Treatment Models for Victims of Violence

Advanced research on trauma treatments indicates that cognitive behavioral approaches are some of the most successful types of treatments.[14,23] Some studies also emphasize the importance of the relational aspects of treatment, such as developing a sense of security and confidence for a positive working relationship between the mental health provider and the patient. Multicultural considerations that influence the perception of the delivery of health services are also important to the success of the treatment.[23] Thus, lay mental health workers (or lay psychosocial community workers (LPCW)) involved in interventions in this project,

must focus on treatments that incorporate cognitive-behavioral strategies based on evidence, are flexible, and that are culturally sensitive.[24]

The fundamental values of treating violence survivors include the empowerment of the survivor and the development of a strong therapeutic alliance between the victim and the mental healthcare provider. This alliance should be flexible, based on mutual respect and culturally sensitive. A standardized format is typically deviated from because it ignores the stressors of everyday life linked specifically to the social circumstances of the survivors of violence, such as being asylum seekers, refugees or internally displaced. An understanding of the psychological suffering caused by violence, neurobiological studies that explain aspects of functional deregulation, and the interaction between culture and symptom expression are also essential parts of the treatment of violence. A focus on holistic treatment based on components is the best way to incorporate all of these concepts.[23]

Starting from an assessment of population characteristics(I don't know how this relates to the second half), the therapeutic intervention may include the following components: psycho-education techniques, relaxation and stress management, modulation of affect, and cognitive processing strategies.[25]

The aim of the psycho-education component is to inform the survivor about the consequences of violence on health, as well to promote understanding of the symptoms they may be experiencing, changes in their behaviors and interpersonal interactions. For many survivors, the ability to understand the symptoms and changes is the beginning of the healing process that allows them to regain their functionality in society. These mechanisms allow for emotional release, emotional support, psychotherapeutic support, and support for self-help which, in turn, help in social adaptation and reduces relapses.

Providing the means for survivors of violence to learn to manage their stress reactions with relaxation techniques is another step to regain mental health. Relaxation techniques aim to break vicious cycles and replace them with a reaction that is compatible with tension reduction. These are behavioral techniques aim at reducing problems such as anxiety, stress or pain. They become part of a systematic desensitization with the purpose of reducing anxiety, because the technique generates self-control over the person's emotional state,

helping to increase the perception of competence, personal domain and the a healthy sleep routine.[26]

The technique for stress management involves controlling and reducing the tension that occurs in stressful situations by making emotional and physical changes. The degree of stress and the desire to make changes both determine the realistic rate of recovery.. The mechanisms to create an individual program for stress management are: to find the positives in situations and not focus on the negatives, plan fun activities, and take regular breaks.[26]

Cognitive strategies highlight the role that perceptions, thoughts and beliefs have on behavior. It is based on several theories, but with a common goal of providing the person with the necessary power to direct or control their own behavior.[23,26]

The ability to contain reactive emotional responses to traumatic stimuli and not feel overwhelmed or burdened by the emotions is an essential part in the process of healing from violence. The conflict between pre-traumatic conceptions of self and the world, such as the belief that "nothing bad will happen to me," and post-traumatic conceptions, such that trauma is evidence that the world is a dangerous place, are contrasted as the survivor perceives the world through the lens of an experience of violence and alters their concept of self and self-esteem. Therefore, restoring the psychological processes of modulating healthy affection and cognitive processes are also important aspects of healing.[25,26]

Given the large number of victims of violence and that Afro-Colombian survivor populations are widely scattered in urban communities and in the smaller towns along the Pacific coast of Colombia, [11,14] a community specific model of health services that could reach these populations will be presented here as an option of treatment.

The World Health Organization (WHO) estimates that from the millions of people suffering from mental health disorders, as much as 76% and 85%, from low and middle income countries respectively, receive no treatment for their disorder.[27] In developed countries, primary care has been called "the cornerstone of community psychiatry." This has promoted the integration and development of skills, knowledge and attitudes of the professional community and society at large.[28]

Due to the legacy of violence and mistrust, the most effective way to reach the Afro-Colombian communities is through civil society organizations. Community-based treatments, consisting of a network of LPCW, allow easy access to victims and, through proposed treatments, generate dynamics that relieve post-traumatic stress symptoms (PTSS).[11]

Interventions based on the community for the treatment of PTSS have been analyzed previously and the tools used allow for the identification treatment that will most effectively improve the quality of life of the individuals.[23,38] However, the methodology should be adjusted to the culture and social characteristics of the victimized population so that the processes develop according to policy actions of do-no-harm. Therefore it is necessary to coordinate studies under the perspective of a specific vision of the population and thus carry out adjustments of treatment tools, in the same way that one should evaluate possible treatments and determine its true efficacy in a sample population to be treated before being used in the entire population.

Cognitive behavioral therapy has been proved effective in different settings for people experiencing different symptoms, especially post-traumatic stress symptoms.[23] The Narrative Therapy model implemented by ACOPLE is grounded on the systematized proposal of Adalberto Barreto, a Brazilian Psychiatrist, Anthropologist and Theologist, that targets to stimulate the construction of solidarity networks of care between people, and strengthen individual and collective resources through socialization and narrative of experiences.[29]

3. OBJECTIVES

3.1. General Objective

To evaluate two community-based mental health interventions designed to decrease symptoms of depression, anxiety, and post-traumatic stress symptoms (PTSS) in Afro-Colombian victims of violence in Buenaventura and Quibdó.

3.2. Specific Objectives

3.2.1. Design two mental health interventions based on LPCW, adapted to socio-cultural characteristics of Afro-Colombian residents in the municipalities of Buenaventura y Quibdó.

3.2.2. Determine the effectiveness of the CETA intervention on mental health designed to diminish symptoms of depression, anxiety, and PTSS in Afro-Colombian victims of violence.

3.2.3. Determine the effectiveness of the NCGT intervention on mental health designed to diminish symptoms of depression, anxiety, and PTSS in Afro-Colombian victims of violence.

4. METHODOLOGY

The methodology of the program was developed under the design, implementation, monitoring, and evaluation (DIME) research model for programs developed and implemented since 2000 by the Applied Mental Health Research -AMHR Group at JHU.

The implementation of this research requires different phases and methodologies that should consider the following phases. The first two phases have already been conducted:

- Phase 1: Qualitative study of the effects of violence.
- Phase 2: Design and validation of instruments based on qualitative data of victims of violence and torture.
- Phase 3: Design and pilot testing of interventions.
- Phase 4: Implementation and evaluation of interventions (Randomized Controlled Trial).

4.1. Phase 1: Qualitative Study of the Effects of Violence

CISALVA Institute staff conducted a pre-intervention qualitative research on the effects of violence on individuals and communities, which included a structured process to identify problems affecting the survivors of violence and their families; JHU staff accompanied the process. The methodology was put in place with a focus on the DIME methodology, which allows a constant monitoring and evaluation process to ensure maximum benefit of the interventions targeted in the study.

Without a relevant process of monitoring and evaluating that is adapted to the local conditions, the standard indicators can be met without this meaning that the needs of the population have been met. In the area of mental health, the ability to monitor and evaluate interventions and programs presents important challenges as the measurements for baseline and post-established treatments must be performed with instruments sensitive enough to identify changes in the behavioral patterns of the population.

This process focused especially on psychological problems that can be caused by violence and the difficulties in the normal operation of a person in society according to the perspective of the interviewee.

It was suggested that interviews be conducted by people who understand the community and belong to AFRODES, which led to the process of implementing the study within the two communities for victims of violence. For this study, 15 people were selected to receive skills training for a qualitative interview for the people in the community.

The interviewers were trained in a variety of qualitative interview techniques (free listing, key respondent interviews, and focus groups) in order to investigate three principal themes: fundamental problems (in particular psychosocial problems) that affect survivors of violence and their families, including how these problems affect the community, their perceived effects, and their causes, and how people face or believe they have to face them. It explored the characteristics of survivors and the activities that people normally do in the society according to the culture, local customs (this last is used to implement adequate measurements of the local level operation). Additionally, the interviewers were trained in reliability of information and ethics employed in relation to the information provided by participants in the study.

Two investigators from CISALVA and three from the Marjorie Kovler Center for Torture Treatment of Heartland Alliance carried out the training of interviewers and coordinated the fieldwork. The duration of the study was two weeks in each city.

The interviewers conducted interviews in pairs. Both for the development of free listing as well as interviews with key respondents, one of the interviewers asked the questions to interviewees and the other handled note-taking.

4.2. Phase 2: Design and Validation of the Instrument Based on Qualitative Data of Victims of Violence and Torture

Using the information obtained in the qualitative study as a base and the identification of the problems that seem to be the most severe, frequent, and of highest importance for the population, existing quantitative measuring instruments were selected and adapted with the purpose of achieving a base-line of the problem faced by victims of violence.

The design was based on a methodology centered on problems prioritized by the community, using interventions derived from the population and appropriately adapted to the expectations of the people, which facilitated their acceptance and understanding, gaining the ability to

more efficiently gather the expected results and later, during the intervention, a new measurement can be taken of new indicators of progress in the community. Thanks to this, it was possible to establish which of the interventions demonstrated successful diminishing of symptoms generated by violent processes.

It was proposed that a modified version of the verification Hopkins Symptoms Checklist (HSCL) be used to evaluate depression and anxiety, and a modified version of the Harvard Trauma Questionnaire (HTQ) be used to evaluate PTSS.[21,22] However, both instruments were modified to determine categories of psychological anguish lived by Afro-Colombian violence survivors.

The survey instruments (HSCL and HTQ) were used to carry out a reference and measurement study of the psychological consequences of violence, including depression, anxiety and PTSS. The evaluations were conducted through direct interviews with at least 120 participants who had either psychological history of sadness, suffering, violence, psychological trauma, or who had no previous symptoms. The result was a picture of the categorization of PTSS, its relative severity in the population of violence survivors, and a reliable instrument with which to measure treatment outcomes.

The investigator team members will carry out the investigation with 15 mental health LPCW (the same individuals in charge of carrying out the qualitative surveys). The collection of data will take approximately two weeks.

The chosen individuals were identified beforehand by AFRODES, under informed consent, as persons that had problems of sadness, suffering, and/or psychological trauma. The LPCW used language identified during the qualitative interviews to formulate the study questions.

The resulting instrument was used in pilot field tests in the cities to evaluate the ease of its use, as well as its level of comprehension between the interviewees. The validity of the local instrument was evaluated through its utilization on recognized groups that have mental health problems originated during violent traumatic events and in recognized groups where this type of problem does not exist. Some participants were interviewed two times by the same or different interviewers to evaluate how reliable the instrument was with the passage of time, and how effective it was when used by different interviewers.

4.3. Phase 3: Design and Pilot Test of Mental Health Intervention

In order to offer mental health interventions based on Afro-Colombian victims of violence, the methodology requires a design and validation process so that the objectives are completed and generate an approach in line with the community problems. To do so, the following aspects have been defined:

4.3.1. Selection and Adaptation of Interventions

Other than identifying primary problems that are going to be served, the qualitative study will also investigate the perceived causes of these problems, their impacts, as well as the ways in which the group confronts these problems and what they feel should or could be done to remedy them. This information will be used to identify and adapt two interventions that may have meaning for Afro-Colombian violence survivors (i.e., interventions that address matters that actually worry them and find solutions to the problems that afflict them) and to be acceptable for them (to be applied in a consistent and appropriate manner with what they now feel).

It is more likely that these interventions include cognitive-behavioral techniques, including the behavioral activation and cognitive processing therapy. At least one of these interventions will be designed with a focus of "components." This method consists in taking into account the actual knowledge about which components of treatment for violence seem to be most efficient and to group them into one intervention. The last studies and opinions of experts suggest that this methodology of treatment for violence has been successful in different contexts; however, it is necessary to adapt them to the scenarios of each culture.[1] Investigators from JHU, in conjunction with the team from CISALVA, will carry out an initial valuation of the literature and of the qualitative investigation results that will drive the design of two specific interventions and the culturally adapted investigation (Interventions A and B) according to the reality of the Afro-Colombian communities.

[1] Preliminary results of JHU Team studies in Northern Iraq suggest that a focus based on components employed there were highly effective.

The JHU and CISALVA personnel will train the supervisors and LPCW so that they can correctly accomplish the chosen interventions. The training sessions will be carried out during a two-week period and will be presented to the LPCW that will carry out the qualitative study.

Afterward, the team of researchers will train two different groups of LPCW in how to deliver the intervention. The training program will provide a basic theoretical foundation about PTSD, mental health, and mental illnesses. It will also provide information about the hierarchies of need and treatment planning, the response to crisis, and the promotion of healthy behavior. The program will provide general directives and abilities of the mental health service features.

4.3.2. Pilot Test of Interventions and Fieldwork

Looking for people affected by violence in the Buenaventura and Quibdó communities requires testing the designed interventions (each intervention against the control group). Therefore, it is also necessary to test the fieldwork through a pilot experience.

The fieldwork pilot will proceed as follows: The AFRODES team will carry out the contacting within the community and perform the informed consent survey. The CISALVA team will make sure that every assessment is completely filled out, entered, and that the information is valid. They will break up the users into groups. The team from HAI will start the interventions while JHU will supervise the attention of the cases and, when the close is finalized the trainers from AFRODES will begin to close the cases. The interviewers from AFRODES will conduct follow up assessments; CISALVA will digitize these assessments and analyze the database.

In order for the intervention pilot tests to work within the communities of Buenaventura and Quibdó, three intervention groups will be designed: Group A will receive the individual intervention adapted by the investigators; Group B will receive the community therapy that is also conditioned based on the groups affected by violence; and the control/waiting list group, whose participants will not receive any intervention but will have an outcome assessment and will get psychological attention offered by HAI. The Groups A and B interventions will be conducted by the LPCW and will be permanently supervised by the project psychologists.

4.4. Phase 4. Randomized Controlled Trial. Implementation and Evaluation of the Mental Health Interventions

4.4.1. Trial Design

A single-blind controlled randomized trial with three parallel groups will be conducted: two intervention groups and one control/waiting list group in each city, powered for comparisons among each intervention against the control group.

4.4.2. Participants

The Participants will come from the municipalities of Quibdó and Buenaventura, located in districts identified as those sheltering large amounts of individuals displaced by the armed conflict in the country (Chocó and Valle del Cauca). Participants will be adults over the age of 18 who are either Afro-Colombians or mestizos referred by key informants (from AFRODES or other community leaders) whom they recognize as having symptoms of depression, sadness or suffering, and having a history of trauma exposure. This will be followed by a snowball technic where the interviewed participants will be asked to suggest others that might have presented the same conditions. Further individuals will be identified, using a door-to-door manner, visiting neighborhoods with high prevalence of victims.

4.4.3. Selection Criteria

4.4.3.1. Inclusion Criteria

In order to select individuals that will likely be a part of the interventions, field visits will be taking place to identity adult (18 years of age or older) Afro-Colombian victims of violence and torture who have expressed sadness, suffering, psychological trauma caused by violence.

Leaders from AFRODES and the CISALVA team will visit the communities accompanying the interviewers to assess the previously identified participants. The surveys will be conducted after participants have been explained to about the study and an informed consent form has been explained and signed with each participant.

The selection of people that will be included in the study will be based on the severity of the symptoms and the level of dysfunction identified using the instruments of the investigation, as

well as the history of exposure to traumatic violent experiences. The inclusion criteria will be: a) being over 18 years of age, b) being Afro-Colombian or mestizo, c) reporting having experienced at least one violent traumatic experience, d) having a TMHS score ≥49, and e) having any reduced functionality in routine activities. Participants' scores will represent a baseline assessment on which to compare with further follow up assessments.

The administrative supervision of the project will be done by the stakeholders: HAI, AFRODES, and CISALVA. Along the research process, the JHU staff will be advisors. The supervision of the fieldwork will be led by three professionals from CISALVA, two professionals from HAI, and the CETA supervision will be led by JHU.

4.4.3.2. Exclusion Criteria

People of ethnicities other than Afro-Colombian or mestizos and children under 18 are not included in the study, therefore they do not fall under the survey inclusion requirements.

Adult Afro-descendants, who show symptoms of severe mental illness like schizophrenia, psychosis, endorsing suicidal thoughts, and those who are a potential harm for others, will be excluded from the study. Excluded individuals due to these criteria will be referred to a psychologist from the study staff to determine the need for specialized psychiatric care; psychologists and social workers will ensure the participant is offered and given appropriate care. Lastly, anyone related to the LPCW will be also excluded.

4.4.4. Description of Interventions

Two interventions will be conducted in a randomized controlled study in Buenaventura and Quibdó. In both cities, there will be a group receiving Intervention A, another receiving Intervention B, and a control/waiting list group to compare each intervention's effectiveness. The wait-list/control group will receive psychological attention once the follow up assessment is conducted.

4.4.4.1. Common Elements Treatment Approach (CETA)

The CETA intervention, which is a transdiagnostic psychotherapy model based on a CBT (Components Based Therapy), was developed by two of the study authors for use in low-

resource countries. CETA components include: (1) Encouraging Participation, (2) Psycho-education, (3) Cognitive coping, (4) Gradual exposure: trauma memories, (5) Cognitive reprocessing, (6) Safety skills, (7) Relaxation, (8) Behavioral activation and (9) Live gradual exposure.

Twenty LPCW (10 from each city) will be trained and supervised via the apprenticeship model of training.[30] They will participate in a 10-day training by CETA experts, followed by weekly practice groups and then personalized supervision meetings with a mental health professional and a local supervisor (one per city) after each session. This will be key, not only to ensure a correct delivery of the model, but also as means to ensure alignment with the Norms and Codes of Practice of Psychology in Colombia.[31]

4.4.4.2. Narrative Community-Based Group Therapy, NCGT

Narrative Therapy is founded on a social construction that puts forth the theory that the stories we tell ourselves and others represent our view of the world and of our relationships; these stories are the most powerful tool we have to modify our lives.[29]

The NCGT consists of a series of steps that will be accomplished throughout 12 sessions of approximately 1 hour and 30 minutes. The first session aims to present the therapy approach and presentation and the last one to close, conclude and evaluate; the 10 sessions in the intermedium aim to conduct the NCGT itself. Therapy sessions will be held in community meeting locations (schools, churches, and community centers). The sessions consist of several steps: 1) Welcome and group dynamics dealt with presentation, ground rules and psycho-education; 2) Sharing and selecting a problematic topic; 3) Adding context to the topic, linkages to suffering; 4) Sharing local knowledge; 5) Closure and relaxation.

In this therapy, the word “narrative” refers to what the person tells him or herself, as well as others, about their lives (dominant history), and the manner in which they have construed symbol and meaning. It also refers to experiential knowledge and how this “local” knowledge has surfaced, how it has been expressed and put together in interactions with others.

The intervention will be directed by LPCW who will be in charge of 5 participants each. The NCGT group will be composed of 25 participants and 5 LPCW, with the supervision and

support of a psychologist and a social worker.

Some of the Techniques of Narrative Therapy:

Naming the Problem: Given a story that is “saturated” or “densely filled” with the problem, a therapist invites the person to put a specific name to the problem. It can be a word or short phrase and allows the person to feel that they are in control of the situation.

Externalizing Language: This consists of the therapist passing onto the a client, in an implicit manner, a language that permits the person to feel that the problem and the effects of the problem over their life, are external instead of the belief that they form a part of the self: “the problem is the problem” and it has nothing to do with the person’s personality or psychological state. Its objective is to help the person to distance themselves from their problems and to consider them as circumstantial problems and interpersonal processes.

Deconstruction – Unexpected Outcomes: Through questions, the therapist invites the person to detail the circumstances and nature of the unexpected outcomes such that they realize how they are out of place with the “saturated” story of the problem, permitting a deconstruction of the dominant story while at the same time allowing for an alternative account that is rich with new meaning.

Use of Therapeutic Documents: The therapist will use records from each group meeting. These documents record achievements, discoveries by the person which relate their progress and alternative accounts.

Use of External Witnesses: The person can choose who to invite to the session and with whom they will share their stories, especially family members, close friends or people in the community with whom they share the same problems. External witnesses can share their own memories with the client in order to reinforce the story with the "resonances" that produced them.

Memory: People can gain support through memories of important people they have lost (through death) or with those with whom they have lost contact such as relatives, friends, neighbors or acquaintances that are highly significant in their lives.

Narrative Therapy is appropriate in community work with those communities that are victims of armed conflict, not only through the concepts already mentioned, but also because it is a therapy based on relationships that requires that people take a stand against the world, that they discover and rediscover themselves, i.e., that they recover their identity. It requires the therapist to be a facilitator of creativity and imagination as they work with metaphors and analogies. This creates an understanding of the complexity of human beings and recognition of the individual experience.

4.4.4.3. Wait-list Control

These participants act as the control group. As intervention participants, they will have a baseline (at the same time as intervention participants) and follow up assessments (about 12 weeks after baseline).

The control participants are unlikely to receive any mental health treatment outside the study as access to these kind of services is extremely limited in both cities.[32] Controls will be contacted by study staff on a monthly basis to screen for acute serious mental problems (suicide ideation, anxiety or depression). When necessary, they will be assessed by a psychologist and if it is needed, they will be excluded from the study and provided with the appropriate care. A psychological evaluation will be offered and provided to each of the controls after their completion of the follow-up and, when necessary, psychological care will be provided in ACOPLE centers.

4.4.5. Enrollment Method and follow up assessment

Allocation of finally accepted participants for either arm of the trial will be done in a randomized fashion by researchers from CISALVA who will not have access to participants. Random assignment will be performed by giving consecutive ID numbers to baseline surveys as they are conducted and these will then be allocated randomly to any of the arms of the trial (CETA, NCGT, wait-list control). The interviewers who will conduct the baseline and follow-up assessments will be masked to the participants' arm allocation. Follow-up assessments for controls will be conducted, approximately, at 3-4 months after baseline assessment according to their time of enrollment.

4.4.6. Training of Participants in the research

4.4.6.1. Lay Psychosocial Community Workers (LPCW)

HAI will carry out the first series of trainings, estimated to be four to six weeks long, about concepts and basic abilities for all the LPCW selected to participate in the project.

The initial plan for the LPCW is based on instructional curriculums used in Iraq and developed for mental health workers with two years of postsecondary education but modified for use in Colombia (see Appendix)

4.4.6.2. Psychologists

Psychologists will be trained by the JHU team and will conduct the training component of the intervention (CETA), according to the schedule and training manual (see Appendix)

Training for NCGT will be conducted by the CISALVA staff and HAI according to the planned program (see Appendix).

4.4.6.3. Interviewers

CISALVA will carry out trainings on assessment instruments. Staff from CISALVA will participate in the training of the interviewers with the support from center coordinators, the field supervisors, and AFRODES leaders.

The training will last three hours and will have the following content:

- Explaining the organic structure of the Project.
- Explaining the security protocol in fieldwork and how to carry out the activity and collection of information (see Appendix).
- Explaining the project and the importance of the survey.
- Survey components of the training, informed consent, and its necessity.
- Validation of learning.

4.4.7. Monitoring and Supervision of the Intervention

4.4.7.1. Administrative Monitoring of the Process:

1. Assistance in the training of Psychologists and LPCW.
2. Monthly progress reports to HAI.
3. Yearly reports to the ethics committee of the Universidad del Valle.
4. Monthly financial reports for the accepted budget under the norms of HAI and the Universidad del Valle.

4.4.7.2. Monitoring of the Intervention Process

CETA Intervention for Group A will have close supervision for all individuals in the study. In each weekly treatment session, the LPCW will keep a tracking form that will include the level of severity of the main symptoms and clinical history for each participant. The LPCW will meet with their local supervisors weekly to discuss each case. Local supervisors will assess the suitability of the tasks performed by the LPCW and provide feedback and support for future sessions according to the case and the LCPW's doubts. Supervisors should answer questions that the LPCW may have regarding identified problems after performing the interventions, as well as the special health needs that they identify in the population (e.g., a person who intends to commit suicide; the supervisor should redirect the person to specific health facilities that can help solve their mental health problems). These adverse events will be reported to the Institutional Review Board of Human Ethics in a format designed for that purpose.

The CETA coordinators and the LPCW will be supervised weekly by phone calls from JHU psychologists, where the intervention as a whole will be evaluated and monitored. Feedback will be provided. At the same time, the supervisors should inform the CISALVA staff about the details and questions that the LPCW team may have regarding the interventions for better support.

The supervisors also have an obligation to deliver all registers and relevant information of the study to the lead researchers from CISALVA. In this way, the researchers can confirm whether the intervention is being carried out correctly and they will be able to identify and correct the difficulties that are presented as the study is carried out. Tracking of session attendance by participants was stored in a google drive account which could only be

accessed by the research team and was secured with a password. To track attendance, participants' codes will be used instead of their names or any other information that could lead to uncovering their identity.

In this group, the supporting documents of the research for CETA will be:

- Baseline assessment.
- Signed informed consent.
- Database baseline assessments.
- Database of random assignment to the intervention Group (A, B, or control).
- Clinical history of each participant.
- Weekly supervision records.
- Document tracking session attendance.
- Follow up assessment.
- Database of follow up assessments.

NCGT Intervention for group B: A close monitoring for all individuals in the study will be conducted. In each weekly session, the LPCW will fill out the tracking record that will include the level of severity of principal symptoms of the person, as well as their clinical history. The LPCW will meet with their local supervisors once per week and will share this information with them. Local supervisors will assess the suitability of the tasks performed by the LPCW and will make suggestions for the further session, based on the current problems of the individual and how he or she responded to previous sessions.

Supervisors should answer questions that the LPCW may have regarding identified problems after performing the interventions, as well as the special health needs that they identify in the population (e.g., a person who intends to commit suicide; the supervisor should redirect the person to specific health facilities that can help solve their mental health problems). These adverse events will be reported to the Institutional Review Board of Human Ethics in a format designed for that purpose.

At the same time, the supervisors should inform CISALVA staff about the details and questions that the LPCW may have regarding the interventions. The supervisors also have an obligation to deliver all registers and relevant information about the study to the principal investigators. In this way, the investigators can confirm whether the intervention is being

carried out correctly and they will be able to identify and correct the difficulties that are presented as the study is carried out. Tracking of session attendance by participants was stored in a google drive account which could only be accessed by the research team and was secured with a password. To track attendance, participants' codes will be used instead of their names or any other information that could lead to uncovering their identity.

In this group, the supporting documents of the research for NCGT will be:

- Baseline assessment.
- Signed informed consent.
- Database baseline assessments.
- Database of random assignment to the intervention Group (A, B, or control).
- Records of each NCGT session.
- Weekly supervision records.
- Document tracking session attendance was stored on Google-Drive.
- Follow up assessment.
- Database of follow up assessments.

Wait-list Control Group: CISALVA researchers will have monthly check in calls with each participant in this group. During the call, participants will be screened for acute serious mental problems (suicide ideation, anxiety or depression). When necessary, they will be assessed by a psychologist and if it is the case they will be excluded from the study and provided with the appropriate care. A psychological evaluation will be offered and provided to each of the controls after their completion of the follow-up and, when necessary, psychological care will be provided in ACOPLE. Participants who decide to drop out from the study (maybe due to adverse events) will be reported accordingly to the Institutional Review Board of Human Ethics for this purpose.

In this group, the supporting documents of the research for the Wait-list Control Group will be:

- Baseline assessment.
- Signed informed consent.
- Database of baseline assessments.
- Database of random assignment to the intervention Group (A, B, or control).
- Follow up assessment.

- Database of follow up assessments.

4.4.8. Definitions of Loss to Follow-up

After randomization is conducted, participants will be allocated to a group (A, B, or Control). During the monitoring, the participants can drop out of the investigation voluntarily, be removed from the study because of particular indications, monitoring failure, or through a deviation from protocol. Definitions are as follows:

- **Voluntary Withdrawal**: Participants from the study who decide not to continue being enrolled in the trial, either for unavailability or whatever other reason. For example, if the person moves to another municipality, suspends the CETA therapy before the management session of the traumatic experience, or suspends the NCGT before the sixth session. Attempts to contact the participant will be done every three (3) weeks, until the end of the study in order to obtain the follow up assessment before declaring loss to follow-up. Only in cases where participants express their desire to abandon the trial, will these participants not be further contacted.
- **Withdrawal by Indication (Adverse Event)**: Includes participants who meet the criteria for withdrawing from the study due to specifically determined circumstances within the protocol like the onset of exclusionary criteria through changes in their clinical state of mental health: psychosis, endorsing suicidal thoughts, and homicidal ideation. In such cases, the participant will be referred to a staff psychologist who will define the need of further specialized care and will offer it to participant if required. These cases will be reported to the Institutional Committee of Human Ethics. These participants will not be followed up with nor will they be included in analysis. In the case of a withdrawn participant showing significantly improved indication, as defined by the staff psychologist, this person could return to the trial. Regarding the follow up assessment, the information will be collected from the participant's clinical record, if it is available.
- **Monitoring Failure**: Includes participants in the study who do not attend the indicated therapy sessions and those for whom it is not possible to obtain monitoring data. Contact attempts will be done every three (3) weeks, until the end of the study, in order to a follow up assessment before declaring the loss to follow up. Definitions for each group are as follow:

- CETA: A participant who has surpassed the trauma experience session (approximately the sixth session) and who discontinues attending therapy sessions and/or whom the study staff fails to re-contact.
- NCGT: A participant who has surpassed the sixth session and discontinues attending therapy sessions and/or whom the study staff fails to re-contact.
- Wait-list Control Group: It is not possible to establish communication with the person to complete the follow up assessment.

- **Protocol Deviation**: Includes patients who, due to different circumstances, do not follow the assigned protocol. These participants would change groups and they will be included in the analyses.

4.4.9. Primary and secondary outcomes

The primary outcomes will be changes in depression, anxiety and post-traumatic stress symptoms.

The secondary outcomes will be changes in the Total Mental Health Symptom (TMHS) and dysfunction scales.

4.4.10. Instrument

A brief instrument validation study was conducted with a sample of Afro-Colombians for the two study communities to evaluate the psychometric properties of the culturally adapted mental health and functional impairment assessments. In addition to measuring mental health problems and functionality, the study questionnaire included socio-demographic and socio-economic items, and questions related to traumatic experiences, access to healthcare services and support networks.[33]

Mental health symptoms will be measured with the Total Mental Health Symptoms (TMHS) scale of 64 items including locally relevant symptoms and sub-scales of depression (n=15 symptoms), anxiety (n=10 symptoms) and PTSD (n=16 symptoms). Depression and anxiety symptoms will be assessed using the Hopkins Symptom Checklist (HSCL-25) and symptoms of trauma using the Harvard Trauma Questionnaire.[34,35] The measures were into Spanish using the language and terminology from the previous qualitative study.[33] The symptom

scales does not measure if a participant has a specific psychiatric diagnosis but rather to characterize severity of the study participant's psychological symptoms.[36,37] The dysfunction measure will be done using a gender-specific questionnaire with 12-item for females and 10-item for males.

For each symptom, respondents will be asked to report the frequency with which they had experienced that symptom in the prior month, with response options: 0 for "never", 1 for "a few times", 2 for "sometimes", and 3 for "all the time". For the dysfunction scale, participants will be asked to state the level of "difficulty" experienced during the previous month in the execution of a named task and included these response options: "no difficulty" scored as 0; "a little difficulty" as 1; "regular" as 2; "a lot" as 3; "cannot execute the task" as 4; "does not apply" as 9. The "does not apply" option was included given that some participants may not perform tasks for reasons other than dysfunction caused by mental health symptoms (e.g. if a question about caring for children was asked to someone who does not have children).

Outcome assessment will be done per individual by the calculation of symptom means for each scale, with symptom scales ranging from 0-3 and dysfunction scales from 0-4.

The survey is comprised of 7 sections (see Appendix):

A. Personal and socio demographic information.
B. Socioeconomic information.
C. Gender-specific dysfunction assessment.
D. Mental health symptoms assessment.
E. Exposure to traumatic experiences information.
F. Ability to address problems and connections.
G. Access to general health care services and mental health services.

4.4.11. Sample Size

The sample size will be calculated taking into account the comparison of two mean scores of the averaged symptoms of each study group with respect to control. Baseline scores will be obtained from the Phase 3 study. Taking into account a difference in averaged changing scores between the control group and the intervention group, with an alpha error of 5%, a power of 80% and the standard deviation obtained.

Using a comparison of two means formula:

$$n = \frac{2\,(Z\alpha + Z\beta)^2 * S^2}{(x_1 - x_2)^2}$$

Where:

- n = subjects needed in each of the samples
- Z_α = Z value corresponding to the desired risk
- Zβ = Z value corresponding to the desired risk
- S^2 = Variance of the quantitative variable the control group has or for reference
- d = Minimum value of the difference to be detected (quantitative data)

Sample size adjusted to losses:

After estimating the possible loss of patients for different reasons (loss of monitoring, abandonment, withdrawal) the sample size will be increased according to such losses. The adjusted sample size according to loss can be calculated as: n (1 / 1–R)

- n = number of subjects not lost
- R = estimated proportion of loss

Taking the average of the worst and the best scenarios (differences in changes between groups of 0.20 and 0.30 points, respectively) and considering that there will be four cycles, the number of subjects will be rounded up to 72. So, to compare interventions A, B with the Wait-list control group, the sample size for each group will be 72 people in the CETA therapy, 72 people receiving NCGT, and 72 people in the Control/Waiting list Group, for a total of 216 people in the Buenaventura investigation and a further 216 in Quibdó.

In order to make it efficient, the individuals that conduct the weekly interventions, CETA therapy or NCGT, where the average duration is between 5 and 12 weeks, it was necessary to program a series of cycles for the collection of sample in each municipality (see Table 2):

Table 2. *Distribution of study participants by intervention group and capacity of participants per Lay Psychosocial Community Workers (LPCW) in each city.*

Capacity of clients per LPCW	CETA	NCGT	Wait-list Control Group	Total clients	Time required

Capacity of clients per LPCW	CETA	NCGT	Wait-list Control Group	Total clients	Time required
Up to 5 people per counselor	24	24	24	72	3-5 months
Up to 5 people per counselor	24	24	24	72	3-5 months
Up to 5 people per counselor	24	24	24	72	3-5 months
Up to 5 people per counselor	24	24	24	72	3-5 months
TOTAL	96	96	96	288	

The total number of required people for the study in both municipalities is approximately 576.

4.4.12. Randomization

Consecutive ID numbers will be assigned to baseline surveys as they are conducted and these will then be allocated to intervention A, B, and to a wait-list control group using a blocked randomization procedure. The allocation sequence will be developed by a statistician from the CISALVA Institute whose participation in the project is limited to the randomization of people into the groups. The results from the interventions will be anonymous as the analysis will be done by an investigator from CISALVA without knowledge of the intervention codes of each participant.

4.4.13. Evaluation of the Psychosocial Interventions

The methodology to be carried out for the comparison of the community interventions will include quantitative and qualitative analyses. The quantitative one will be done through a controlled trial that is random, single-blind, longitudinal and prospective.

4.4.13.1. Quantitative Impact of the Intervention

There is little evidence on the effectiveness of psychosocial interventions in the target population. Assessments of pre and post-intervention changes in non-controlled studies are not sufficient to demonstrate the effectiveness as other factors may also have an effect on peoples' outcomes. This control group usually improves symptoms over time, even without any kind of intervention; the latter change can be due to the mere passage of time or a regression to the average among those that were identified as having severe problems at the baseline. Keeping this natural tendency in mind, each of the interventions will be effective if only those that receive the intervention are evaluated. To demonstrate the real effect, a control group should be assessed wherever possible and any change in this group must be subtracted from the changes found among those receiving the intervention. The resulting difference is the only real measurement of intervention impact.

For the purpose of this trial, it is proposed that the methods used in other settings be adopted: two interventions will be evaluated in two separate tests carried out simultaneously in the study cities (one in Buenaventura and the other in Quibdó). Within each test the people that are eligible for the study (based on the screening evaluation and the quantitative instrument) will be divided into two groups: those who receive the intervention (whether through CETA or NCGT), and those who will be allocated to the wait-list control group.

To assess effectiveness of the interventions, a follow up assessment will be conducted two (2) weeks after the end of each intervention and after 3-4 months after baseline assessment for those in the wait-list group. If an intervention group had significantly better changes (from baseline to follow up assessments) than the control group, the intervention will be considered effective.

Baseline characteristics of participants from intervention and control groups will be compared using Chi-square tests or Fisher's exact test for categorical variables and t tests or Wilcoxon test for continuous variables. The main indicators of effect will derive from a comparison of the mean changes scale scores from baseline to follow-up assessment between intervention and control groups. End-point analysis, where baseline scores are carried forward as follow-up assessment scores, will be used in cases where the team fails to re-contact some participants; a widely used method in mental health clinical trial,[38] thereby assuming no change over the course of study period for these individuals. An Intention-to-treat sample will be used to mitigate potential selection bias by participants or interviewers.

Additionally, a descriptive analysis of the demographic variables and those related to mental health will be carried out for each arm. The quantitative variables will be described according to statistics of central tendencies and dispersion, and the qualitative variables according to frequency. The analyses of different groups will be conducted in accordance to tests of significant statistics using the sampling distributions of the variables.

The quantitative and qualitative analyses of the effectiveness of the interventions will take place approximately three months after the base line. This time is approximate because it will depend on the inclusion rate of subjects in the study. The effectiveness of the intervention is estimated to significantly reduce the general symptoms generated by violence on the mental health of the individuals after three months, as has been observed in other previous studies.

4.4.13.2. Qualitative Impact of the Intervention

A qualitative evaluation will be performed of the interventions from material recorded in the transcripts of the CETA and NCGT therapies. This qualitative analysis will allow a description of unexpected effects, both negative and positive, of the interventions, as well as the perceptions of study participants, as feedback to better the formulation and delivery of interventions. Results from these analyses will also permit to validate in a qualitative way the study so that strategic partners from ACOPLE can advocate on its behalf to scale-up the methodologies, if effective.

4.4.14. Statistical Analysis Plan.

For the analyses, the outcome will be defined as the average score among items of a specific scale (e.g., depression, anxiety). The analysis approach will use longitudinal mixed effects regression models assessing interaction of study arm (any intervention [i.e. CETA or NCGT] vs. the control group) with time. Thus, the interaction is a measure of the difference in changing scores over time (follow-up minus baseline) between intervention and control. Mixed effects regressions will be estimated separately for each city (Buenaventura and Quibdó). The approach for handling missing data is to apply multiple imputation techniques for all missing values. Lost to follow up will be handled using two methods: 1) multiply imputing scores for those lost to follow up and 2) a sensitivity analysis of creating an inverse probability weight of follow up and including this weight as a covariate in the final analysis.

4.4.14.1. Multiple imputation

Missing data will be imputed using multiple imputation procedures. All variables will be imputed, with exception of (somewhat) static demographic variables at follow-up (e.g. age, gender, education). All cluster variables above the individual level (e.g. counselor, cycle, etc.) will be predictors of imputations with imputing equations using legal ranges. Multiple imputation procedures will be performed separately for each city (Buenaventura and Quibdó) and for each trial (CETA vs. control and NCGT vs. control) databases.

All missing values of a particular scale will be imputed. For missing data both at baseline (T0) and follow-up (T1), an iterative process will be used for imputing both T0 and T1 using other T0 and T1 variables as predictors, and then replacing imputed values at T0 with only T0 predictors, for avoiding temporal incoherence among imputed variables. The full multiply imputed database will be used for the Intention to Treat Analysis (ITT), according to the subject's arm allocation after the randomization.

4.4.14.2. Selection of covariates for final mixed models

Potential confounders will be identified, in imputed datasets, by means of two strategies:

1) Baseline differences between groups (CETA vs. control; NCGT vs. control).
2) Potential confounders of changes on scales outcomes over time.

The first strategy will use baseline characteristics and scale scores to explore what's different across groups – (CETA vs. control; NCGT vs. control) – by means of appropriated statistical tests (e.g. T-tests, Chi-squared, Fisher's exact, etc.). Anything significantly different at a 0.10 level between treatment (CETA or NCGT) and control, will be included as a covariate in final mixed models. When a baseline outcome score is significantly different between groups, we will not include as a covariate when looking at that same outcome in final models.

In the second strategy, covariates associated with changes in scale outcomes over time will be identified using mixed (xtmixed) models with an interaction between the potential confounder and time, as fixed effects. If the variable of interest is associated with change in the scale outcome at the 0.10 level, then it will be included the final ITT model. Also, all socio-demographic variables (i.e. gender, age, marital status, education/employment status) will be

included as covariates in the ITT models. Clustering variables (LPCW or cycle) will be included as random effects.

Finally, all possible confounding variables (both dichotomous and continuous) will be centered, in order to report the averaged sample effect of the treatment, CETA or NCGT, among the sample of subjects of each trial, in each city (Buenaventura or Quibdó).

4.4.14.3. ITT Final Mixed Models

Full imputed datasets will be organized as a longitudinal array with time assessments clustered within study subjects, who are clustered within LPCW (for the CETA trial) or within recruitment cycle (for the NCGT trial). Analysis will be performed with the information of both cities but also split by city (Buenaventura and Quibdó); in the lasts models the city will not be included as a clustering variable. Treatment effects are defined as the difference of differences between intervention and control between time intervals. Thus, treatment effects will be expressed as the interaction term between time and treatment, controlling by all identified covariates in the mixed effects regressions (xtmixed). We will include number of weeks at follow-up as a covariate if it was associated at the $p < 0.10$ level for each scale outcome. Clustering variables will be included as random effects. The mixed models will use the identity link function. Imputation procedures are imputing baseline missing values and follow-up missing values, so each person who is missing follow-up will have their follow-up scores imputed.

4.4.14.4. Subgroups analyses

For each study trial (CETA vs. control and NCGT vs. control), in each city, the following subgroups analyses will be done:

- Females subsample.
- Males subsample.
- Non-married/non-coupled subjects subsample.
- Married/coupled subjects subsample.

4.4.14.5. Sensitivity Analyses with Follow-up Predictors

Results of ITT analyses will be tested by sensitivity analyses adjusting for the conditional probability of nonresponse at follow-up. Thus, the baseline covariates and baseline symptom/function scores will be used for predicting the follow-up by means of simple logistic regressions clustered by lay community worker, in the CETA trial, or by recruitment cycle, in the NCGT trial. Variables associated with follow up at an alpha level of 0.10 will be used. If no variables in the data set were predictive of loss to follow up, then we will assume that data missing due to loss-to-follow up is missing completely at random (MCAR). Using the prediction from results of logistic regressions, the inverse of the probability of follow-up will be calculated. The inverse gives us the conditional probability of non-response at follow-up: the "iweight" variable.

The distribution of iweight will be explored using histograms. If outliers appeared, they will be winsorized bringing only the outliers in the higher end of the tail (p=0.01). We will use the inverse weight as a covariate in the mixed (xtmixed) regression models of follow-up sensitivity analyses.

4.4.14.6. Sensitivity Analyses excluding regression outliers

Per each scale outcome, regression outliers will be identified using the residuals from ITT mixed models. Outliers will be identified by looking at numbers above/below an absolute value of 4 standard deviations against the expected number according to the mixed model. Sensitivity analyses will be done excluding those outliers, in order to test the robustness of ITT regressions results.

4.4.14.7. Effect Sizes

It will be done using the Cohen's D approach to effect size calculation. The numerator will be the difference of difference between intervention and control between time intervals, per each outcome (i.e. specific mental health symptoms and dysfunction). Denominator will be the pooled standard deviation of each outcome at baseline, across levels (i.e. clusters) of the model in the non-imputed dataset.

4.5. Ethical considerations

The inclusion of individuals in the study poses minimum risk for both participants and interviewers. The information that will be solicited from the individuals is related to their attitudes and thoughts towards the situations and problems that affect their lives and to how they believe these problems can be solved.

The community based intervention has produced benefits in the majority of the populations where it has been implemented; however there is a risk that interventions can produce unexpected negative results like those that occur with local or national interventions by government attention programs. In the case this were to happen, provisions have been put forth that will allow the interventions to be stopped to avoid any type of damage to the population.

In order for a person to be included in the study, the person must give their consent by signing an informed consent agreement (see Appendix) which clarifies all the pertinent information about the study and how the information given by the participants will be used. People's participation in the study is completely voluntary and every participant can withdraw from the study at any point. The collected information will not be used under any circumstance for other purposes not related to the objectives of this study.

Additionally, in case any participant suffers intimidation or is at risk due to threats by actors outside the study, the center and study will provide appropriate support in getting competent authorities to manage the risky situation. Moreover, when a risky situation presents, any fieldwork activity will be suspended to secure the lives and integrity of any person.

Personal data from individuals, other than name, identification and contact information, will not be collected. In the case there is need to use participants' information for purposes outside the aims of this study, it would be necessary to request special permission from the ethics committee at Universidad del Valle.

The safety of the study staff (researchers, field staff and interviewers) is of equal priority. For this reason, it has been decided that interviewers should come from the same community so they can understand the dynamics of the area serve and do some sort of risk assessment in any given situation. At the least sign of risk to the integrity or life of the interviewers or study staff, the work will be suspended and activities will not resume until there is assurance that

there is no further risk for the group. In the same manner, interviewers will work in groups of at least 5 people and will wear outfits and nametags that identify them as study personnel for security reasons. The objectives and methodology of the study will be presented to community leaders and people from the communities in order to increase awareness of our project to increase safety by the time fieldwork starts.

The researchers of the study, due to the fact that they are people from outside the community, will direct the fieldwork from installations in the Seminar Building of San Buenaventura, which lies outside the fieldwork zone, and in the city of Quibdó they will stay in a hotel that is located across from a police station. This will ensure that the researchers are at minimum risk while the study is carried out. In case threats are received or in any other moment where there may be evidence that the group is at risk, any activity will be suspended and only restarted when the situation is addressed and safety concerns are adequate.

This research proposal was developed in accordance with the legal guidelines of resolution 8430 of 1993 of the Republic of Colombia. Finally, the study was approved by the Institutional Review Board of Human Ethics, CIREH (from the Spanish acronym), of Universidad del Valle, Colombia with code 014-011.

5. TIMETABLE

	2011											2012												2013								
**Activity**	Feb	Mar	Apr	May	Jun	Jul	Aug	Sep	Oct	Nov	Dec	Jan	Feb	Mar	Apr	May	Jun	Jul	Aug	Sep	Oct	Nov	Dec	Jan	Feb	Mar	Apr	May	Jun	Jul	Aug	Sep
Project presentation to the IRB	■														■																	
Lay Psychosocial Community Workers (LPCW) identification		■																														
Training of researchers in qualitative methods		■	■																													
Qualitative study			■	■																												
Analysis of the Qualitative study (QS)				■																												
Modification to the survey based on QS				■																												
Training of interviewers about the interventions				■																												
Pilot test of the survey					■	■																										
Analysis of pilot test of the survey						■																										
Selection and definition of psychosocial interventions (PI)						■	■	■	■																							

	2011											2012												2013								
**Activity**	Feb	Mar	Apr	May	Jun	Jul	Aug	Sep	Oct	Nov	Dec	Jan	Feb	Mar	Apr	May	Jun	Jul	Aug	Sep	Oct	Nov	Dec	Jan	Feb	Mar	Apr	May	Jun	Jul	Aug	Sep
Staff training on PI of the trial																																
Staff training on Informed consent and the survey																																
Pilot test of the trial																																
Adjustments to interventions after the pilot test																																
Selection, baseline assessment, and randomization of 1st cycle of clients																																
1st cycle of intervention																																
Follow up assessment (1rs cycle)																																
Selection, baseline assessment, and randomization of 2nd cycle																																
2nd cycle of intervention																																
Follow up assessment																																

	2011											2012												2013								
**Activity**	Feb	Mar	Apr	May	Jun	Jul	Aug	Sep	Oct	Nov	Dec	Jan	Feb	Mar	Apr	May	Jun	Jul	Aug	Sep	Oct	Nov	Dec	Jan	Feb	Mar	Apr	May	Jun	Jul	Aug	Sep
(2nd cycle)																																
Selection, baseline assessment, and randomization of 3rd cycle																																
3rd cycle of intervention																																
Follow up assessment (3rd cycle)																																
Selection, baseline assessment, and randomization of 4th cycle of clients																																
4th cycle of intervention																																
Follow up assessment (4th cycle)																																
Data Analysis																																
Writing report with results and discussion																																
Delivery of Final Report to IRB and HAI																																

6. REFERENCES.

1. Murray L, Dorsey S, Bolton P, et al. Building capacity in mental health interventions in low resource countries: an apprenticeship model for training local providers. *Int J Ment Health Syst.* 2011;5(1):30.
2. United Nations High Commissioner for Refugees (UNHCR). [International Year for Afrodescendant People]. 2011:4. http://www.acnur.org/t3/fileadmin/scripts/doc.php?file=t3/fileadmin/Documentos/RefugiadosAmericas/Colombia/Situacion_Colombia_-_Afrodescendientes_2011. Accessed Sep 2011.
3. International Council of Swedish Industry (NIR), Asociación Nacional de Empresarios en Colombia (ANDI), Fundación Ideas para la Paz (FIP). [Complex Environments: Buenaventura]. 2011:70. http://cdn.ideaspaz.org/media/website/document/52eac7346a293.pdf. Accessed Sep 2011.
4. Presidential Agency for Social Action and International Cooperation (Acción Social). [Dynamics of Forced Displacement (June 2010)]. In: National Observatory of Forced Displacement, ed. Bogota D.C.: Presidency of the Republic of Colombia; 2010:15.
5. Superior School of Public Administration / Escuela Superior de Administración Pública (ESAP). *[Territorial diagnosis].* Quibdó: ESAP, Sistema de Documentación e Información Municipal; 2001.
6. Cisalva Institute. Profile: Municipality of Quibdo. 2006:10. http://prevencionviolencia.univalle.edu.co/observatorios/choco/quibdo/archivos/perfil_quibdo.pdf. Accessed Sep 2011.
7. Amnesty International. *Amnesty International’s briefing to the UN Committee against Torture on the Republic of Colombia.* London: Amnesty International; 2003.
8. Colombian Coalition against Torture. *Alternative Report to the 4th Periodic Report of the Colombian State to the Committee Against Torture.* Bogota D.C.: Colombian Coalition against Torture; 2009.

9. Presidential Agency for Social Action and International Cooperation (Acción Social). *[Register of Displaced Population].* Bogota D.C.: Acción Social; 2009.

10. Heartland Alliance for Human Needs and Human Rights, Instituto Cisalva, Johns Hopkins University, Asociación de Afrocolombianos Desplazados - AFRODES. Analysis of mental health services in Buenaventura and Quibdo. Chicago: Heartland Alliance for Human Needs and Human Rights; 2009.

11. Gerrity E, Keane T, Tuma F. *The Mental Health Consequences of Torture*. New York: Springer Science & Business Media; 2001.

12. Danelius H. *Convention against Torture and Other Cruel, Inhuman or Degrading Treatment or Punishment.* New York: UN; 2008.

13. American Psychiatric Association. *Diagnostic and Statistical Manual of Mental Disorders, Fourth Edition: DSM-IV-TR®*: American Psychiatric Association; 2000.

14. Sjolund BH, Kastrup M, Montgomery E, Persson AL. Rehabilitating torture survivors. *J Rehabil Med.* 2009;41(9):689-696.

15. Keller A, Lhewa D, Rosenfeld B, et al. Traumatic experiences and psychological distress in an urban refugee population seeking treatment services. *J Nerv Ment Dis.* 2006;194(3):188-194.

16. Keller AS. Caring and advocating for victims of torture. *Lancet.* Dec 2002;360 Suppl:s55-56.

17. United Nations Office for the Coordination of Humanitarian Affairs (OCHA). Internal Displacement - Overview. http://www.unocha.org/what-we-do/advocacy/thematic-campaigns/internal-displacement/overview. Accessed Sep 2011.

18. United Nations High Commissioner for Refugees (UNHCR). On the Run in Their Own Land. *Internally Displaced People* 2011; http://www.unhcr.org/pages/49c3646c146.html. Accessed Sep 2011.

19. Perez-Olmos I, Fernandez-Pineres PE, Rodado-Fuentes S. [The prevalence of war-related post-traumatic stress disorder in children from Cundinamarca, Colombia]. *Rev Salud Publica (Bogota).* 2005;7(3):268-280.

20. Pineda DA, Guerrero OL, Pinilla ML, Estupinan M. [Usefulness of a screening questionnaire for post traumatic stress in a Colombian population]. *Rev Neurol.* 2002;34(10):911-916.
21. Richards A, Ospina-Duque J, Barrera-Valencia M, et al. Posttraumatic stress disorder, anxiety and depression symptoms, and psychosocial treatment needs in Colombians internally displaced by armed conflict: A mixed-method evaluation. *Psychol Trauma.* 2011;3(4):384-393.
22. Sistiva-Castro DL, Sabatier C. Violence sociopolitique, ESPT et Coping religieux: Une étude comparative en Colombie. / Socio-political violence, posttraumatic stress, and religion as a coping strategy. A comparative study in Colombia. *Revue Francophone du Stress et du Trauma.* 2005;5(2):97-107.
23. Bisson J, Andrew M. Psychological treatment of post-traumatic stress disorder (PTSD). *Cochrane Database Syst Rev.* 2007;18(3):CD003388.
24. Bass J, Bolton P, Murray L. Do not forget culture when studying mental health. *Lancet.* 2007;370(9591):918-919.
25. Kinzie J. Psychotherapy for massively traumatized refugees: the therapist variable. *Am J Psychother.* 2001;55(4):475-490.
26. Johns Hopkins University Bloomberg School of Public Health, Cisalva Institute, Heartland Alliance International. Protocol of Cognitive-Behavioral Therapy Based on Components. Community Care Program for Victims of Violence. Cali: Universidad del Valle; 2011.
27. Funk M, Drew N, Freeman M, Faydi E, World Health Organization (WHO). Mental health and development: Targeting people with mental health conditions as a vulnerable group. Italy: WHO; 2010: http://whqlibdoc.who.int/publications/2010/9789241563949_eng.pdf?ua=1. Accessed Feb 2011.
28. World Health Organization (WHO). *Psychiatry and primary health care. Report on a working group.* Geneva: WHO;1973.
29. Barreto AdP. *Terapia comunitária passo a passo*. Brazil: Gráfica LCR; 2005.
30. Murray L, Dorsey S. Intervention: Colombian version. Therapist manual. Cali: Heartland Allaince International; 2011.

31. Congress of Colombia. Act 1090 2006 (Wednesday September 6) whereby the exercise of the profession of psychology is regulated, dictates the Code of Ethics and Bioethics and other provisions. 46.383 ed. Bogota D.C.: Congress of Colombia; 2006.
32. Heartland Alliance International. *Treating survivors in Colombia.* Chicago: Heartland Alliance International; 2011.
33. Santaella J, Bonilla-Escobar FJ. Validating an instrument for victims of violence in Colombia. *[Unpublished data].* 2011.
34. Derogatis LR, Lipman RS, Rickels K, Uhlenhuth EH, Covi L. The Hopkins Symptom Checklist (HSCL): a self-report symptom inventory. *Behav Sci.* 1974;19(1):1-15.
35. Mollica R, Caspi-Yavin Y, Bollini P, Truong T, Tor S, Lavelle J. The Harvard Trauma Questionnaire. Validating a cross-cultural instrument for measuring torture, trauma, and posttraumatic stress disorder in Indochinese refugees. *J Nerv Ment Dis.* 1992;180(2):111-116.
36. Chorpita BF, Daleiden EL. Mapping evidence-based treatments for children and adolescents: application of the distillation and matching model to 615 treatments from 322 randomized trials. *J Consult Clin Psychol.* 2009;77(3):566-579.
37. Barlow DH, Farchione TJ, Fairholme CP, et al. *Unified protocol for transdiagnostic treatment of emotional disorders: Therapist guide*: Oxford University Press; 2010.
38. Bolton P, Bass J, Neugebauer R, et al. Group interpersonal psychotherapy for depression in rural Uganda: a randomized controlled trial. *JAMA.* 2003;289(23):3117-3124.

LIST OF APPENDICES

6.1. Appendix No 1. Survey

A. Survey of the study

Interviewer Number ________

Respondent Number: _________

Date: DD/MM/YY: ___________

Start Time____________ **End Time** ____________

Name of the person who recommended the interviewee: ____________________________

SECTION A: DEMOGRAPHICS

A1. Gender:	______ Male ______ Female
A2. What is your age:	_______ years
A3. What is your Marital status	_____ Single<br>_____ Married<br>_____ Divorced<br>_____ Separated<br>_____ Widowed
A4. Education	_____ Primary School<br>_____ High School<br>_____ University
A5. Have you been displaced?<br><br>A5a. If the answer is yes, are you registered at Accion Social or other similar organization?	___ Yes ___NO<br><br>___ Yes ___ NO
A6. Do you have any disabilities?<br><br>A6a. If the answer is yes, what kind of disability?	___ Yes ___NO<br><br>_____________________________
A7, What is your current work situation?	___ Full time job<br>___ Part time job<br>___ Self employed<br>___ Not working

SECTION B: SOCIO ECONOMIC

B1. How many people help with the home expenses in your family?	_____ Number
B2. How many people economically depend on	

you?	_____ Number
B3. Characteristic of the house you live in	___ Own<br>___ Rented<br>___ Loaned<br>___ Invaded
B4. Type of home	___ House<br>___ Apartment<br>___ Vicinity<br>___ Campsite<br>___ Shelter
B5 What are your house walls predominantly build of?	___ Plastic<br>___ Wood<br>___ Cardboard<br>___ Other<br>___________ Specify
B6. What is your house floor predominantly build of?	___ Mosaic<br>___ Polished Concrete<br>___ Concrete<br>___ Dirt<br>___ Wood<br>___ Other:<br>___________ Specify
B7. How many people sleep per room?	_____ Number
B8. What kind of social security do you have?	___ Contributory<br>___ Subsidized<br>___ None<br>___ Other ___________ Specify

SECTION C: FUNCIONALITY

For each task say: In the last 4 weeks, how much difficulty have you had doing

FUNCTIONS IN WOMEN	Amount of difficulty doing the task-activity					
**Tasks/Activities**	None	Little	Moderate amount	A lot	Often cannot do	Not applicable
CM01. to work (Q)	0	1	2	3	4	9
CM02. To clean oneself (Q)	0	1	2	3	4	9
CM03. Organize my life (Q)	0	1	2	3	4	9
CM04. To take care of oneself	0	1	2	3	4	9

health/go to the doctor (Q)						
CM05, To take care of the children (Q)	0	1	2	3	4	9
CM06. To do domestic work (Q)	0	1	2	3	4	9
CM07. To give love to family members (Q)	0	1	2	3	4	9
CM08. To keep the home unified (Q)	0	1	2	3	4	9
CM9. To belong to support groups (Q)	0	1	2	3	4	9
CM10. To show oneself as an example to the community (Q)	0	1	2	3	4	9
CM11. To respect others when having meeting with the community (Q)	0	1	2	3	4	9
CM12. To socialize with neighbors (Q)	0	1	2	3	4	9

For each task say: In the last 4 weeks, how much difficulty have you had doing

FUNCTIONS IN MEN	Amount of difficulty doing the task/activity					
**Tasks/activities**	None	Little	Moderate amount	A lot	Often cannot do	Not applicable
CH01. To play sports/to exercise (Q)	0	1	2	3	4	9
CH02. To clean oneself (Q)	0	1	2	3	4	9
CH03. To clean the common areas near the house (Q)	0	1	2	3	4	9
CH04. To work (Q)	0	1	2	3	4	9
CH05. To dialogue with own children (Q)	0	1	2	3	4	9
CH06. To keep the family together (Q)	0	1	2	3	4	9
CH07. To take care of the children (Q)	0	1	2	3	4	9
CH08. To participate in activities of the community (Q)	0	1	2	3	4	9
CH09. To beware of the necessities of the community and help to find solutions for the problems of the community	0	1	2	3	4	9
CH10. To collaborate with the neighbors to find solutions to the conflicts of the community	0	1	2	3	4	9

SECTION D: MENTAL HEALTH SYMPTOMS

The following questions are about some problems that you may or may not have experienced. Please answer YES or NO according to what you think:

**Problems**	**No**	**Yes**
D01. Do you think you dealing with a problem of being sad most of the time?	0	1
D02. Do you think you dealing with a problem of being suffering or frightening most of the time?	0	1
D03. Do you think you dealing with a problem of psychological trauma most of the time?	0	1

The following questions are about some symptoms you may or may not have experienced. For each please tell how often you have felt in the past four weeks: a lot, moderate, a little bit, not at all.

For each symptom say: For the problem _____ in the last 4 weeks, how often have you experienced it.

**Problems**	Not at all	A little bit	Moderate amount.	A lot
D04. Feeling low in energy, slowed down (Hopkins Symptom Checklist – Depression [HSCL-D], Qualitative Symptoms [Q])	0	1	2	3
D05. Blaming yourself for things (HSCL-D)	0	1	2	3
D06. Crying easily (HSCL-D, Q)	0	1	2	3
D07. Loss of sexual interest or pleasure (HSCL-D)	0	1	2	3
D08. Poor appetite (HSCL-D, Q)	0	1	2	3
D09. Difficulty falling asleep, staying asleep (HSCL-D, Harvard Trauma Questionnaire [HTQ], Q)	0	1	2	3
D10. Feeling hopeless about the future (HSCL-D, HTQ, Q)	0	1	2	3
D11. Feeling sad (HSCL-D, Q)	0	1	2	3
D12. Feeling lonely (HSCL-D, Q)	0	1	2	3
D13. Thoughts of ending your life (HSCL-D, Q)	0	1	2	3
D14. Feeling of being trapped or caught (HSCL-D, Q)	0	1	2	3
D15. Worrying too much about things (HSCL-D, Q)	0	1	2	3
D16. Feeling no interest in things (HSCL-D, Q)	0	1	2	3
D17. Feeling everything is an effort (HSCL-D)	0	1	2	3
D18. Feelings of worthlessness (HSCL-D, Q)	0	1	2	3
D19. Suddenly scared for no reason (Hopkins Symptom Checklist – Anxiety [HSCL-A], Q)	0	1	2	3

D20. Feeling fearful (HSCL-A, General Symptoms [GS], Q)	0	1	2	3
D21. Faintness, dizziness or weakness (HSCL-A, GS, Q)	0	1	2	3
D22. Nervousness or shakiness inside (HSCL-A, GS, Q)	0	1	2	3
D23. Heart pounding or racing (HSCL-A, GS, Q)	0	1	2	3
D24. Trembling (HSCL-A, GS, Q)	0	1	2	3
D25. Feeling tense or keyed up (HSCL-A, Q)	0	1	2	3
D26. Headaches (HSCL-A, GS, Q)	0	1	2	3
D27. Spells of terror or panic (HSCL-A, Q)	0	1	2	3
D28. Feeling restless, can't sit still (HSCL-A)	0	1	2	3
D29. Repeated disturbing memories, thoughts or images of an event (HTQ, Q)	0	1	2	3
D30. Repeated disturbing dreams of an event (HTQ)	0	1	2	3
D31. Suddenly acting or feeling as if event were happening again/reliving it (HTQ)	0	1	2	3
D32. Feeling very upset when something reminds you of event (HTQ, Q)	0	1	2	3
D33. Having physical reactions (heart pounding, breathing, sweating) when something reminds you of event (HTQ, GS, Q)	0	1	2	3
D34. Avoiding thinking about or talking about event or avoiding having feelings related to it (HTQ, Q)	0	1	2	3
D35. Avoiding activities or situations because they remind you of event (HTQ, Q)	0	1	2	3
D36. Trouble remembering important parts of event (HTQ, Q)	0	1	2	3
D37. Loss of interest in activities you used to enjoy (HTQ)	0	1	2	3
D38. Feeling distant or cut off from other people (HTQ, Q)	0	1	2	3
D39. Feeling emotionally numb or being unable to have loving feeling s for those close to you (HTQ, Q)	0	1	2	3
D40. Feeling irritable or having angry outbursts (HTQ, Q)	0	1	2	3
D41, Having difficulty concentrating (HTQ, Q)	0	1	2	3
D42. Being 'super-alert' or watchful or on guard (HTQ, Q)	0	1	2	3
D43. Feeling jumpy or easily started (HTQ, Q)	0	1	2	3
D44. Accelerated breathing (dyspnea) or asphyxiated (GS)	0	1	2	3
D45. Chest pain (GS)	0	1	2	3
D46. Weird feeling about oneself or of unreality (GS)	0	1	2	3
D47. Numb and tickles (GS)	0	1	2	3
D48. Blush and chills (GS)	0	1	2	3

D49. People talking incoherence (Q)	0	1	2	3
D50. People laugh, scream or sing in a euphoric way (Q)	0	1	2	3
D51. People talk euphorically with other people (Q)	0	1	2	3
D52. Fear to get out of the house or to talk to other people (Q)	0	1	2	3
D53. Anxiety attack (Q)	0	1	2	3
D54. Loss of color in the face and brightness in the eyes (Q)	0	1	2	3
D55. Loss of happiness (Q)	0	1	2	3
D56. People do not trust other people anymore (Q)	0	1	2	3
D57. I feel I am being followed (Q)	0	1	2	3
D58. Pain in the soul (Q)	0	1	2	3
D59. Loss of self-steam (Q)	0	1	2	3
D60. Anxiety, cannot control oneself (Q)	0	1	2	3
D61. Use drugs or alcohol to find relief for symptoms (Q)	0	1	2	3
D62. I feel intolerant at other people (Q)	0	1	2	3
D63. Loss of memory, people cannot remember anything (Q)	0	1	2	3
D64. I feel impotent, as I cannot do anything to solve my problems (Q)	0	1	2	3
D65. I feel unstable (Q)	0	1	2	3
D66. I have feeling to take revenge (Q)	0	1	2	3
D67. I feel angry about certain things in my life (Q)	0	1	2	3
D68. I have violent daydreams and fantasies against people that bother me (Q)	0	1	2	3

SECTION T: Traumatic Experiences

Please respond if you have suffered or seen that it happened to someone else any of the following situations:

**Situations**	
T01. Have a member of your family or a neighbor been disappeared or killed by armed groups in control of a region?	0 No 1 Yes
T02. Threats, humiliation or someone has been hurt to make it run away or cause damage	0 No 1 Yes
T03. Have you been kicked or beaten with a bat, a stick or other objects?	0 No 1 Yes
T04. Have you been forced to watch how a person is tortured or killed?	0 No 1 Yes
T05. Attacked from armed groups?	0 No 1 Yes
T06. Have you being rape or sexually abused?	0 No 1 Yes
T07. Have you witness the assassination of a person?	0 No 1 Yes

T08. Displacement, loss of home and or property caused by pressure by an arm group?	0 No 1 Yes
T09. Other traumatic events<br>Specify___________	0 No 1 Yes

SECTION E. COPING, SERVICE USAGE AND SOCIAL CONNECTIONS

In the next questions I will ask you how often you do different things to help yourself when you feel bad. These are different things that some people do. You may do some also or you may not do them. For each one, please tell whether you do it often, sometimes, rarely or not at all to help yourself feel better.

	Not at all	Rarely	Sometimes	Often
E01. Go to church (Q)	0	1	2	3
E02. Find psychological help from a professional (Q)	0	1	2	3
E03. To think in a positive way (Q)	0	1	2	3
E04. Visit the justice house (Q)	0	1	2	3
E05. Seek neighbors for help (Q)	0	1	2	3
E06. To find strength and to strive (Q)	0	1	2	3
E07. Look for friends to talk about the problems (Q)	0	1	2	3
E08. To get spiritual help (Q)	0	1	2	3
E9. To get help from social organizations (Q)	0	1	2	3
E10. Participate in recreational activities with the community (Q)	0	1	2	3
E11. To accept reality as it is (Q)	0	1	2	3
E12. Be patient (Q)	0	1	2	3
E13. Watch TV or do other things to take my mind off my problems (Q)	0	1	2	3

Now, I am going to ask you about questions about your relationships with other people. Please tell me No or Yes in agreement of what you think:

Relationships	No	Yes
E14. I am happy with the friendships I have.	0	1
D15. I feel I belong in my community.	0	1
E16. I know people who will listen and understand me when I need to talk.	0	1
E17. I have people that I am comfortable talking with about my family's problems.	0	1

E18. In a crisis, I would have the support I need from family or friends.	0	1

The next three questions ask about how often you socially connect with other people. By "socially connect," we mean social interactions you have with other people. Examples of social interactions are playing sports, doing religious activities, taking courses, having discussions or eating meals together.

	> 8 times	5-7 times	2-4 times	0-1 times
E19. In the last 2 weeks, how often did you socially connect with your friend?	0	1	2	
E20. In the last 2 weeks, how often did you socially connect with other families from the community?	0	1	2	3

I am going to ask you about relationships with people in the community who are not in your family.

	Never	Rarely	Sometimes	Often
E21. In the last month, how often have people visited you in your home?	0	1	2	3
E22. In the last month, how often have you visited people in their home?	0	1	2	3
E23. If you suddenly needed a small amount of money, for example like enough to pay for your household for one week, how many people could you turn to who would be willing to provide this money?				Number
E24. If you suddenly faced a long-term emergency, such as a family death or harvest failure, how many people could you turn to who would be willing to assist you?				Number

Now we want to learn more generally about the different types of services you may have used in the past 6 months. For each type of service, I will ask whether you have used it in the past 6 months

**Services**	No	Yes
E25. Have you gone to the health clinic for your own health problems?	0	1
E26. Have you receive psychosocial services	0	1
E26a. If yes, what did you receive and from whom?: ____		
E27. Have you received any non-money assistance (e.g. animal, farm materials)?	0	1
E27a. If yes, what did you receive?: ____		
E28. Are you currently participating in any loan or grant activities?	0	1

We would also like to ask you several questions about your general level of satisfaction with different components of your life. What is your level of dissatisfaction-satisfaction concerning?

	**None**	**A Little bit**	**Regular**	**Very good**	**Excellent**
E29. Your health	0	1	2	3	4
E30. Your financial situation	0	1	2	3	4
E31. Your housing	0	1	2	3	4
E32. Your life in general	0	1	2	3	4

Documents provided upon request.

6.2. Appendix No 2. Informed Consent

6.3. Appendix No 3. Manual of Common Elements Treatment Approach (CETA)

6.4. Appendix No 4. Manual of Narrative Community-Based Group Therapy (NCGT)

6.5. Appendix No 5. Participant Referral Form for Adverse Events

6.6. Appendix No 6. Security Protocol Manual
